# Model-based extrapolation of ecological systems under future climate scenarios: The example of *Ixodes ricinus* ticks

**DOI:** 10.1371/journal.pone.0267196

**Published:** 2022-04-22

**Authors:** Henning Nolzen, Katharina Brugger, Adam Reichold, Jonas Brock, Martin Lange, Hans-Hermann Thulke

**Affiliations:** 1 Department of Ecological Modelling, Helmholtz-Centre for Environmental Research GmbH–UFZ, Leipzig, Germany; 2 Unit for Veterinary Public Health and Epidemiology, University of Veterinary Medicine Vienna, Vienna, Austria; 3 Competence Center for Climate and Health, Austrian Public Health Institute (Gesundheit Österreich), Vienna, Austria; Universiti Teknologi Malaysia, MALAYSIA

## Abstract

Models can be applied to extrapolate consequences of climate change for complex ecological systems in the future. The acknowledged systems’ behaviour at present is projected into the future considering climate projection data. Such an approach can be used to addresses the future activity and density of the castor bean tick *Ixodes ricinus*, the most widespread tick species in Europe. It is an important vector of pathogens causing Lyme borreliosis and tick-borne encephalitis. The population dynamics depend on several biotic and abiotic factors. Such complexity makes it difficult to predict the future dynamics and density of *I*. *ricinus* and associated health risk for humans. The objective of this study is to force ecological models with high-resolution climate projection data to extrapolate *I*. *ricinus* tick density and activity patterns into the future. We used climate projection data of temperature, precipitation, and relative humidity for the period 1971–2099 from 15 different climate models. Tick activity was investigated using a climate-driven cohort-based population model. We simulated the seasonal population dynamics using climate data between 1971 and 2099 and observed weather data since 1949 at a specific location in southern Germany. We evaluated derived quantities of local tick ecology, e.g. the maximum questing activity of the nymphal stage. Furthermore, we predicted spatial density changes by extrapolating a German-wide tick density model. We compared the tick density of the reference period (1971–2000) with the counter-factual densities under the near-term scenario (2012–2041), mid-term scenario (2050–2079) and long-term scenario (2070–2099). We found that the nymphal questing peak would shift towards early seasons of the year. Also, we found high spatial heterogeneity across Germany, with predicted hotspots of up to 2,000 nymphs per 100 m^2^ and coldspots with constant density. As our results suggest extreme changes in tick behaviour and density, we discuss why caution is needed when extrapolating climate data-driven models into the distant future when data on future climate drive the model projection.

## Introduction

A major challenge of our time is to cope with the impacts of anthropogenic climate change. It is evident that there will be huge negative consequences for humans and nature [1]. However, for many ecological systems it is still unclear how climate change will affect them due to the high degree of complexity and numerous interactions.

The castor bean tick *Ixodes ricinus* is the most widespread tick species in Europe and especially in Germany [2]. It is an important vector of pathogens causing Lyme borreliosis and tick-borne encephalitis and is therefore highly relevant from the perspective of public health [3–5]. The life cycle of *I*. *ricinus* ticks consists of four life cycle stages: eggs, larvae, nymphs and adults. Once the eggs have hatched into larvae, each further life cycle stage requires a blood meal from a host to develop to the next life cycle stage [3]. For this purpose, *I*. *ricinus* becomes active and climbs up the vegetation to wait there with outstretched feelers for a host to attach itself to. The seeking of a host is also called questing.

In recent years, the geographical distribution of *I*. *ricinus* in Sweden and Norway has shifted further north and additional alterations are expected [6, 7]. Moreover, shifts towards higher altitudes have taken place in European mountain regions as a possible result of increasing temperatures [8–11]. Gray et al. [12] describe an increase of the tick population due to an extended questing season caused by mild winters. On the other hand, changing precipitation patterns may lead to an increased mortality of ticks due to droughts [13]. Nonetheless, the understanding of the future number of ticks in a given area (i.e. the abundance) and temporal activity patterns (i.e. the times when ticks are active) is far from complete [12–15].

Reliable predictions of future nymphal densities are generally difficult because tick population dynamics, which encompasses a multi-year life cycle, depend on complex, interacting biotic and abiotic factors such as temperature, relative humidity, precipitation, habitat type, and host availability [14, 15]. A better understanding of future *I*. *ricinus* population development is nevertheless desirable, e.g. because a shift of the activity period of ticks would also shift the risk profile of an infectious tick bite [12].

Seasonal questing activity patterns of ticks inform about the timing of tick bites in regions where ticks occur, whereas tick density or density estimation informs about potential risk areas for tick bites. In general, activity is considered as strongly influenced by temperature, precipitation, and relative humidity [16–18]. This is because *I*. *ricinus* ticks need a certain temperature range to start activity and a humid micro-climate with a relative humidity of at least 80% to survive [19].

Models can be applied to predict the seasonal tick population dynamics of the next year based on tick density and other biotic and abiotic predictors of past years [15]. Alkishe et al. [20] modelled the ecological niche of *I*. *ricinus* to inform about the geographic distribution with respect to current climate. These models use climate variables as input data. Hence it appears logical to force the model with climate scenarios. All other things being equal, the climate-driven tick population dynamics known at present is projected into the future using variables from climate models [21]. The more detailed and fine-grained available climate predictions are, the more promising is the application of tick population modelling to future climate scenarios. The comparison of past and present system behaviour supports understanding of possible climate change related vulnerabilities of the ecological system and potential adverse effects for health and biodiversity [22].

As part of the European branch of the EURO-CORDEX initiative [23], new high-resolution climate data (e.g. temperature, precipitation, humidity, and wind speed) has recently become available for the European continent. The data set has a horizontal grid resolution of 0.11 degree (~12.5 km) and is available for the period 1971–2099. This refined scale facilitates capturing local impacts of climate change on small-scale ecological systems sensitive to local conditions [24]. Since the dispersal range of ticks is limited, they are a good example to study the effects of such small scale changes using high-resolution climate data. At lower spatial resolution, for example 100 km x 100 km [25], the climate variation between different habitat types (forests, grasslands, urban areas) cannot be considered.

The objective of this study is to force two ecological climate-sensitive models of *I*. *ricinus* with high-resolution climate projections. We want to assess how associated health risks due to predicted density and activity patterns change in line with different climate change scenarios. We take the overall tick activity and density as a proxy for the risk of being bitten by an infected tick, because it is a simple method of estimating the risk of tick-borne diseases and both models have no Lyme disease component. Additionally, we discuss whether the extrapolation of tick behaviour observed in the present and under today’s climatic conditions into a distant future has practical applications.

## Materials and methods

### Data

We considered the following climate parameters: daily near-surface mean air temperature, daily near-surface minimum air temperature, daily near-surface maximum air temperature and the daily mean of relative humidity. We used time series for these climate parameters for the period 1971–2099 from the latest available high-resolution climate model simulations and time series of observed weather data for the available period 1949–2020. We linked these time series to a rule-based tick population model. With this we investigated the resulting peak nymphal questing activity, the nymphal questing activity during each of the four seasons of a year, and the minimum nymphal summer activity for past, present, and future years until 2099 for a specific location in southern Germany. Further on, we linked these meteorological time series to a statistical forecasting model from Brugger et al. [17] to assess the spatial impact on the density of *I*. *ricinus* across Germany under three scenarios (2012–2041, 2050–2079, 2070–2099) compared to the reference period (1971–2000). In the following, we describe the source and content of the climate simulation data and observed weather data used as model inputs.

#### Observed tick densities

In principle, the models can be used for any region as long as daily meteorological data is available. Here, we selected the particular region around a sampling site in Haselmühl (11.8819° E, 49.4083° N, altitude 430 m). For this sampling site, monthly data on nymphal tick densities (i.e. questing nymphs per 100 m^2^) is available for the period 2009–2018 according to Brugger et al. [15, 18] and G. Dobler (pers. comm).

#### Observed weather data

From the German Weather Service (DWD) we used daily temperature and humidity data for the period between 1949 and 2020 [26]. In particular, the following data sets were used from the weather station in Regensburg (Station ID: 4104; Location: 12.1023° E, 49.0426° N, altitude 365 m):

- Daily mean of temperature at 2 m height [°C]
- Daily minimum of air temperature at 2 m height [°C]
- Daily maximum of air temperature at 2 m height [°C]
- Daily mean of relative humidity [%]

Since there is a 65 m difference between the weather station (365 m), and the sampling site in Haselmühl (430 m), the temperature values from the DWD data were adjusted according to the dry-adiabatic temperature gradient (e.g. [27]). Here we applied a correction factor of -0.64°C = -0.98°C * (430 m—365 m) / 100 m.

#### Projected climate data

We used climate simulation data covering Germany with a horizontal grid resolution of 0.11 degree (~12.5 km). A total of 15 combinations of Global Change Models (GCMs) and Regional Change Models (RCMs) were provided by The Climate Service Center Germany (GERICS) for the RCP8.5 scenario, i.e. the scenario that exceeds 4K global warming, with the r1i1p1 driving ensemble, respectively the r3i1p1 ensemble for the Max-Planck-Institute for Meteorology (MPI-M) models (Table 1). We used bias-adjusted EURO-CORDEX simulation data [23] of

- daily near-surface air temperature [K]
- daily maximum near-surface air temperature [K]
- daily minimum near-surface air temperature [K]

and homogenised EURO-CORDEX simulation data for

- the near-surface relative humidity [%].

**Table 1:** Overview of EURO-CORDEX simulations used as model input.

Institute	Driving global climate model (GCM)	Downscaling regional climate model (RCM)	Reference period 1971–2000	Near-term scenario (1.5 K) 2012–2041	Mid-term scenario (3.0 K) 2050–2079	Long-term scenario (4.0 K) 2070–2099
Canadian Centre for Climate Modelling and Analysis (CCCma)	CanESM2	CCLM4-8-17 (v1)	8.6 ± 0.7	9.8 ± 0.7	11.9 ± 1.0	13.2 ± 0.9
		REMO2015 (v1)	8.6 ± 0.7	9.7 ± 0.7	11.4 ± 0.9	12.4 ± 0.9
Institute Pierre Simon Laplace (IPSL)	IPSL-CM5A-MR	WRF381P (v1)	8.6 ± 0.7	10.1 ± 0.6	11.7 ± 0.8	12.5 ± 0.6
		RACMO22E (v1)	8.6 ± 0.9	9.9 ± 0.6	11.8 ± 0.8	12.6 ± 0.7
		RCA4 (v1)	8.6 ± 1.0	9.8 ± 0.5	11.6 ± 0.8	12.4 ± 0.8
Met Office Hadley Centre (MOHC)	HadGEM2 ES	ALADIN63 (v1)	8.6 ± 0.9	10.4 ± 0.9	12.1 ± 0.9	13.4 ± 1.0
		HIRHAM5 (v2)	8.6 ± 0.7	10.3 ± 0.8	12.1 ± 0.9	13.3 ± 0.9
		REMO2015 (v1)	8.6 ± 0.9	10.3 ± 0.9	11.9 ± 0.9	13.4 ± 1.1
		RegCM4-6 (v1)	8.6 ± 0.8	10.2 ± 0.8	12.1 ± 0.8	13.3 ± 0.8
		WRF381P (v1)	8.6 ± 0.8	10.1 ± 1.1	11.7 ± 1.0	12.7 ± 0.9
		RACMO22E (v2)	8.6 ± 0.9	10.3 ± 0.8	12.3 ± 0.9	13.5 ± 0.9
		HadREM3-GA7-05 (v1)	8.6 ± 0.8	10.6 ± 0.8	12.6 ± 1.0	13.9 ± 1.0
		RCA4 (v1)	8.6 ± 0.9	10.2 ± 0.9	11.8 ± 0.9	13.1 ± 1.0
Max-Planck-Institute for Meteorology (MPI-M)	ESM-LR	REMO2015 (v1)	8.6 ± 0.7	10.0 ± 0.5	10.9 ± 0.8	11.9 ± 0.7
		RCA4 (v1)	8.6 ± 0.6	9.8 ± 0.6	11.1 ± 0.8	12.1 ± 0.7

For the 15 combinations of Global Change Models (GCM) and Regional Change Models (RCM) the mean annual temperature in Germany for four periods are given. All simulations were run with the RCP8.5 scenario.

Bias adjustment is a post-processing method to identify and adjust for possible biases between observed and simulated climate variables of the RCM output [28, 29]. Homogenisation means that irregularities in the climate data caused by non-climatic influence factors such as the relocation of weather stations or the changing of measuring instruments are detected and corrected to improve the overall quality [30, 31]. The data set was provided in netCDF format, a standard format used for exchange of climate data [32]. We extracted the meteorological time series for the years 1971 to 2099. Temperature variables were converted from Kelvin to Celsius (°C = K—273.15). The raster cell of the EURO-CORDEX climate simulation data corresponding to the tick sampling location in Haselmühl is defined by the following coordinates (longitude, latitude): 11.8220° E, 49.4827° N (top left corner), 11.9907° E, 49.4918° N (top right corner), 11.8361° E, 49.3731° N (bottom left corner), 12.0044° E, 49.3821° N (bottom right corner). It has an average altitude of 425 m according to the SRTM Digital Terrain Model of Germany [33] and hence no adiabatic adjustment was required. A map of the sampling site in Haselmühl can be found in Fig 1 in Brugger et al. [18].

**Fig 1:**
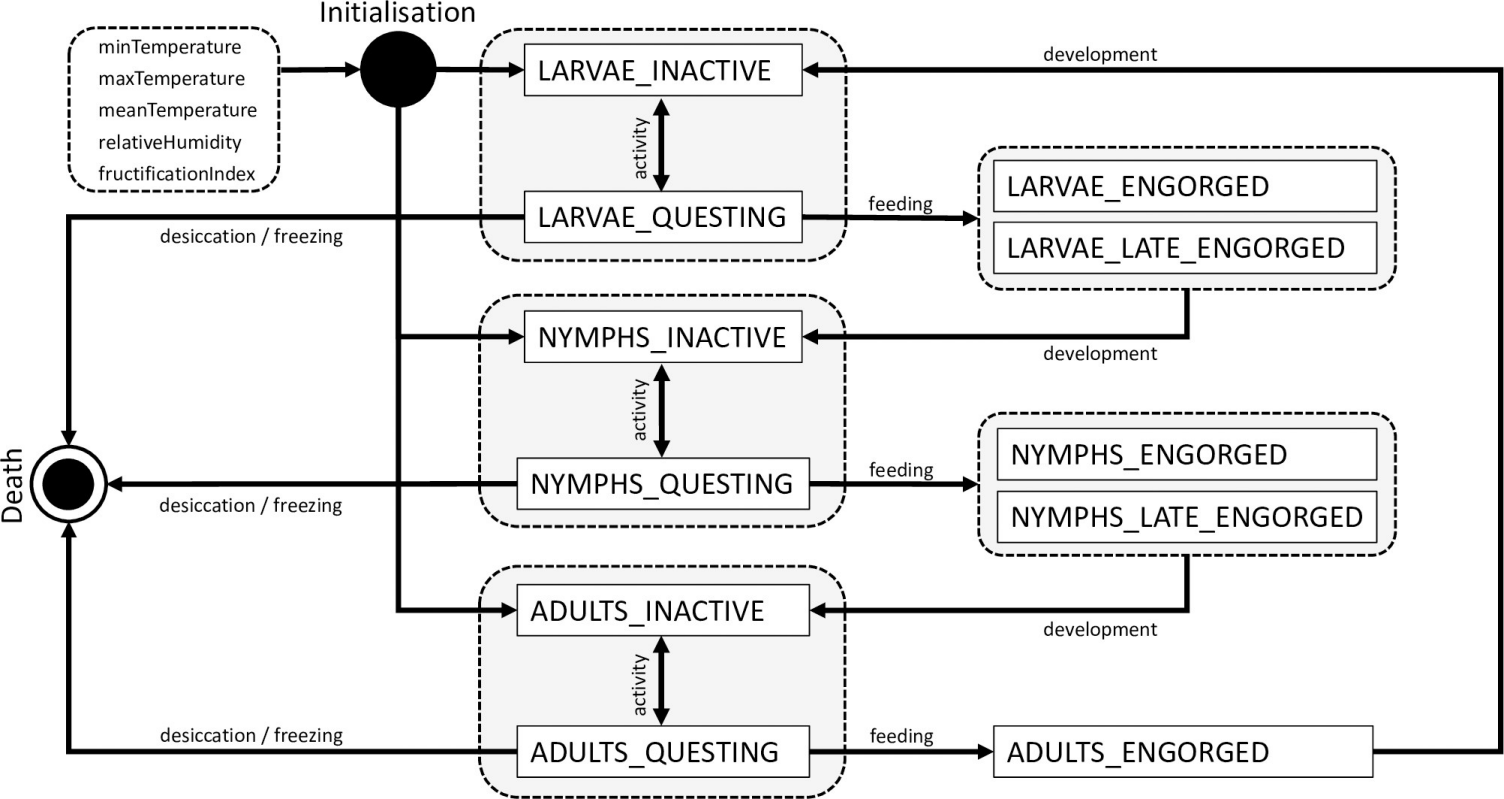
State transition diagram of the IRIS model (Ixodes RIcinus Simulator). The model simulates the development process of *I*. *ricinus* from larvae to nymph to adult stages forced by climate conditions. The white boxes represent the model cohorts. The black arrows indicate the transition between cohorts, which is controlled by the respective model process indicated by its label.

### Local tick population model

#### Model description

We implemented IRIS (*Ixodes RIcinus* Simulator), a spatially-explicit, cohort-based population model to simulate the activity of *I*. *ricinus* ticks. It is driven by daily mean, minimum and maximum temperature, and relative humidity. The model landscape consists of grid cells that each have one of three idealised habitat types–forest, meadow and ecotone (i.e. the transition zone)–which represent different environmental conditions.

Per grid cell of the landscape a stochastic cohort model of tick life-cycle stages is implemented. Possible transitions between stages and questing activity are simulated with daily time steps over one year using daily meteorological data as input. Fig 1 shows the state transition diagram of the model. It represents the following schedule of basic processes of a tick life cycle: (i) host seeking, i.e. questing or inactivation of ticks, (ii) development to the next life stage, and (iii) mortality by desiccation or freezing.

We do not explicitly model the egg life stage because our model only covers one year. It is assumed that the number of new larvae depends on the number of engorged adult ticks. The natural mortality of adult ticks is realised by them leaving their cohort when they lay eggs.

**Fig 2:**
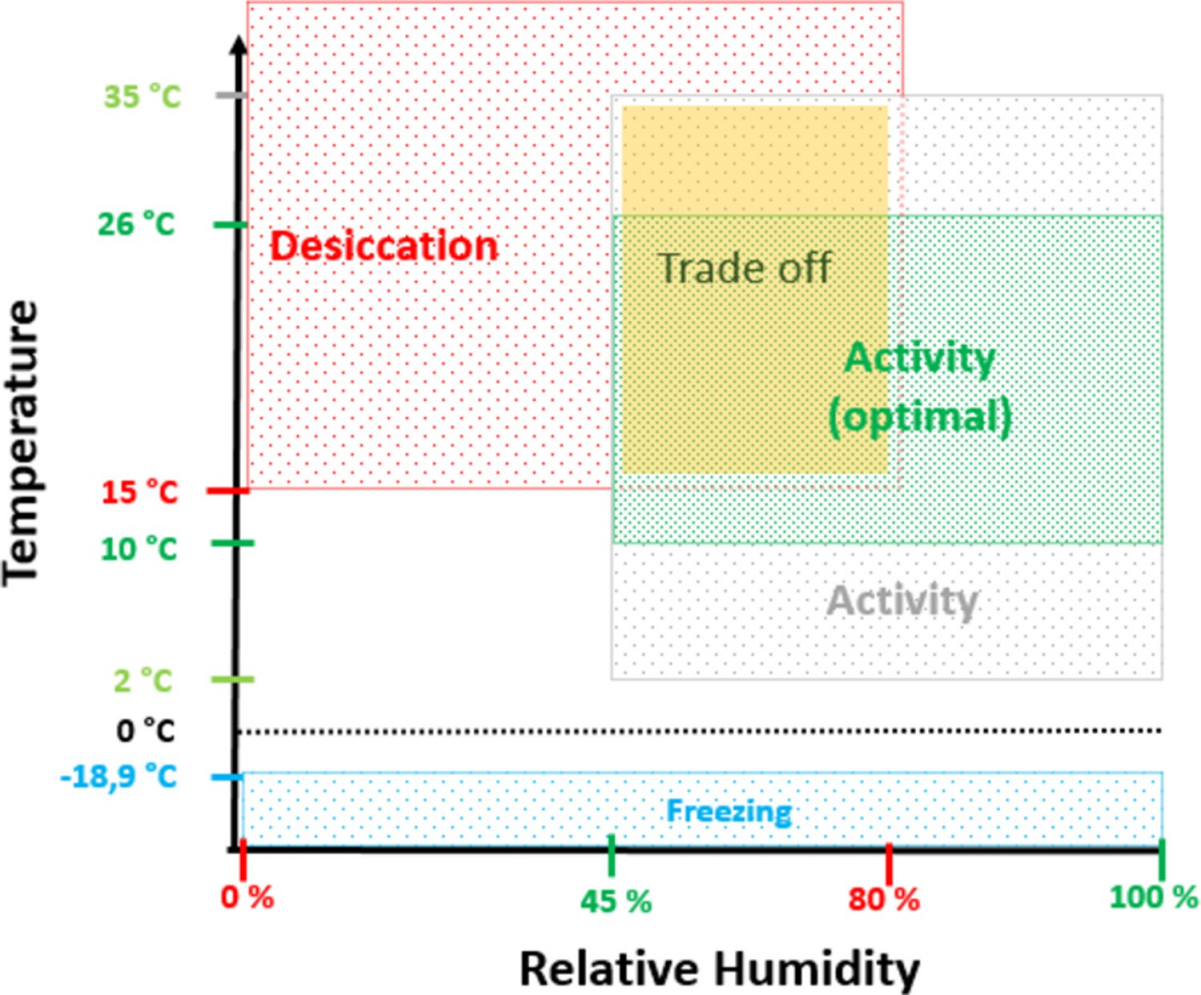
Trigger values of the climate parameters and the induced change of behaviour state of the ticks. Optimal conditions for tick activity is given in green, conditions leading to desiccation or freezing in red and blue, respectively. The overlap between the areas admitting activity and implying desiccation illustrates the trade off each individual tick faces when deciding whether to become active for host-seeking or to stay inactive to conserve humidity (indicated in yellow).

The cohort dynamics are driven by the temperature and relative humidity. Fig 2 shows the trigger values of the climatic parameters and the induced change of behaviour state of the ticks. A References for the respective values can be found in S1 Appendix.

A complete model description following the ODD protocol (Overview, Design concepts, Details), a standardised scheme for describing process-based simulation models [34, 35], can be found in S1 Appendix. The model was written in Java. The source code is online available online under https://git.ufz.de/ecoepi/iris. An archived copy of the repository with the model version we used to simulate the results can also be found under DOI:10.5281/zenodo.5948986.

#### Model calibration

**Fig 3:**
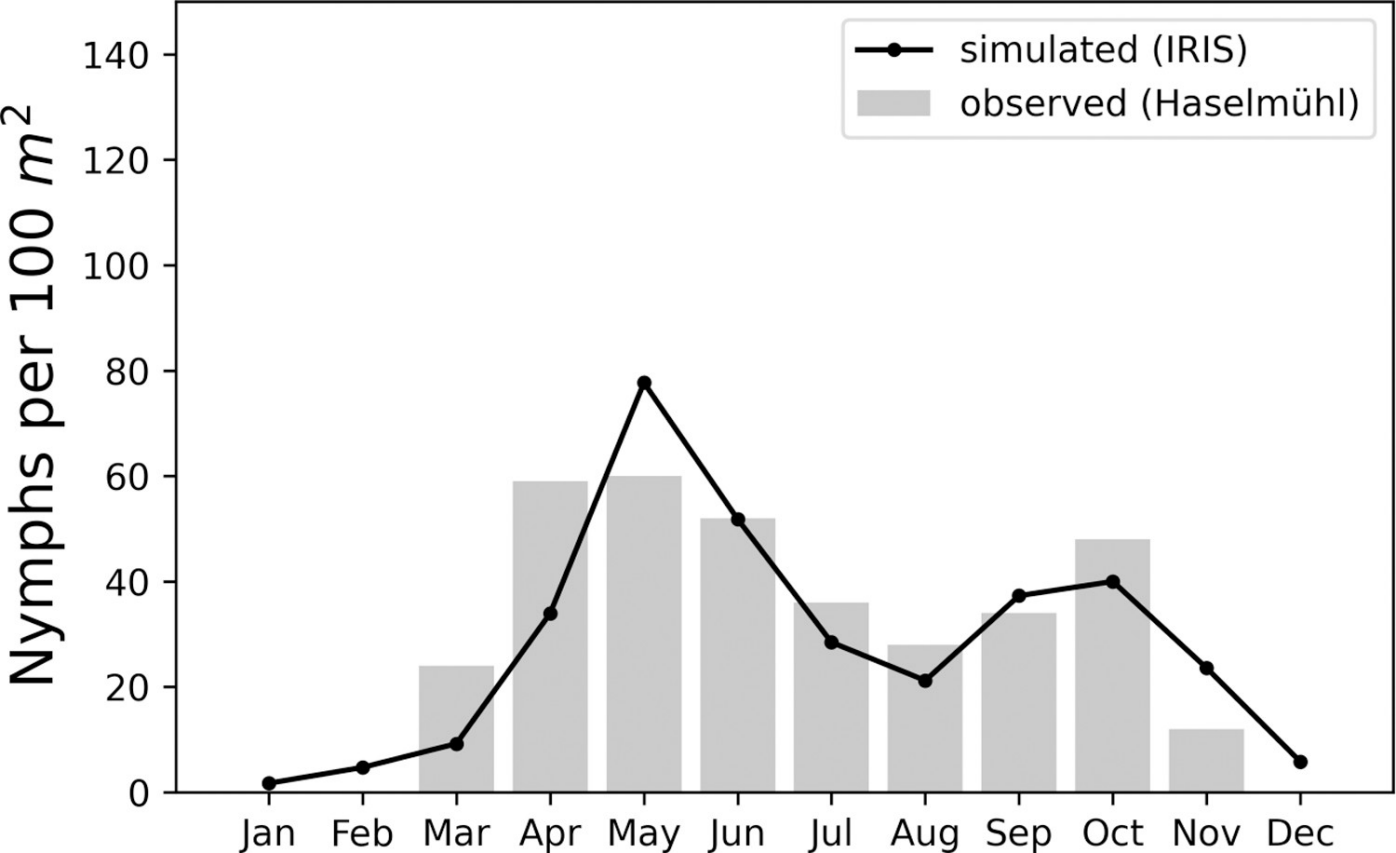
Result of the model calibration for the year 2013. The grey bars show the monthly observed nymphal densities (nymphs / 100 m^2^) at the sampling site in Haselmühl. The black connected dots show the monthly simulated nymphal densities with calibrated parameter values. The root-mean-square error (RMSE) is 11.29.

To calibrate the IRIS model we performed a systematic parameter variation for the activation rate and the initial number of ticks. We selected the combination of parameter values that minimised the difference between the number of nymphs simulated by the model and the observed number of nymphs in Haselmühl for the years 2009 to 2018. We used the root-mean-square error (RMSE) as measure for the difference between simulated and observed data. With the calibrated parameters we ran the model and recorded the number of active nymphs over the year. We determined the month with the highest nymphal activity and compared it to the observed month with the highest nymphal activity at the sampling site in Haselmühl. This comparison showed good agreement with the observed peaks of nymphal activity. An illustrative example of the comparison for the year 2013 can be found in Fig 3. Detailed results of the calibration for the years 2009 to 2018 can be found in S2 Appendix.

#### Simulation experiments

Every simulation run of the IRIS model covered one year between 1971 and 2099 using the respective time series for mean, minimum and maximum temperature, and relative humidity from one of the 15 different climate models, i.e. GCM-RCM combinations. This resulted in 129 times 15 = 1935 simulations runs of daily numbers of questing nymphs. Further, we ran the model with observed weather data from the DWD weather station in Regensburg for the years between 1949 and 2020, i.e. 72 simulation runs of daily numbers of nymphs.

#### Model analysis

From the simulation output we derived three quantities of the time-dependent nymph activity. These were contrasted with metrics characterising the temperature of individual years or seasons.

*a) Derived quantities* *describing tick dynamics*. First, the calendar day with maximum number of active (questing) nymphs in the simulation landscape was recorded from the annual simulations. The descriptor addresses the time of peak activity of nymphs. Secondly, the seasonal density of active nymphs was determined according to the meteorological calendar. The aim was to identify the seasons with pronounced or less important changes in nymph activity along the climate projection horizon. Third, the minimum density of questing nymphs during summer months (June to August) was determined from the annual simulations. The longer periods with hot and dry weather are in a year, the less nymphs will remain active at the end of the period, and consequently the density of active nymphs is expected to be minimal.

*b)* *Climate related metrics*. For each year the median temperature was calculated either for the full year or the early seasons only (January to June), as well as the annual median temperature of the year. Alternatively, the running year between 1949 till 2020, and 1971 till 2099 was used as possible indicator of intensifying climate change.

We used the median temperature of the years to limit the impact of phases with abrupt temperature extremes. In the IRIS model, it depends on how often the temperature threshold is reached or exceeded, but it is neglected how far a respective temperature threshold is exceeded. This corresponds to the median because it is frequency-dependent instead of magnitude-dependent, which is why we used it as the independent variable of the descriptor. Since the model is insensitive to outliers, the descriptor should be accordingly. For reference, we have included the derived quantities with the annual mean temperature, the annual median temperature, and the years in S3 Appendix.

### German-wide tick density model

We used the negative binomial regression model introduced by Brugger et al. [17] to compare georeferenced projections of spatial nymphal density of *I*. *ricinus* across Germany. The nymphal density is estimated using mean annual temperature, mean temperature of driest quarter, annual precipitation, mean annual relative humidity, land cover/use, and geographical latitude.

For comparison with the reference period (0K, 1971–2000) three climate scenarios were considered: the near-term scenario (1.5K, 2012–2041), the mid-term scenario (3K, 2050–2079), and the long-term scenario (4K, 2070–2099). The 30-year intervals were selected symmetrically around the years in which mean temperatures first reached plus 1.5K, 3K, 4K, i.e. 2026, 2064, and 2084 (Table 1).

For the climate scenarios, 30-years means of the bioclimatic parameters were calculated with the daily values of the EURO-CORDEX simulation data set (Table 1). The land cover/use was classified using the CORINE (Coordination of Information on the Environment) dataset of the European Environment Agency [36]. Analogous to Brugger et al. [17], we defined five main classes: agricultural areas, broad-leaved forest, coniferous forest, mixed-forest, and urban areas. Contrary to the climate parameters, predictions on land cover/use were not available. Therefore, they were assumed to be constant for the three scenarios of the climatic future.

**Fig 4:**
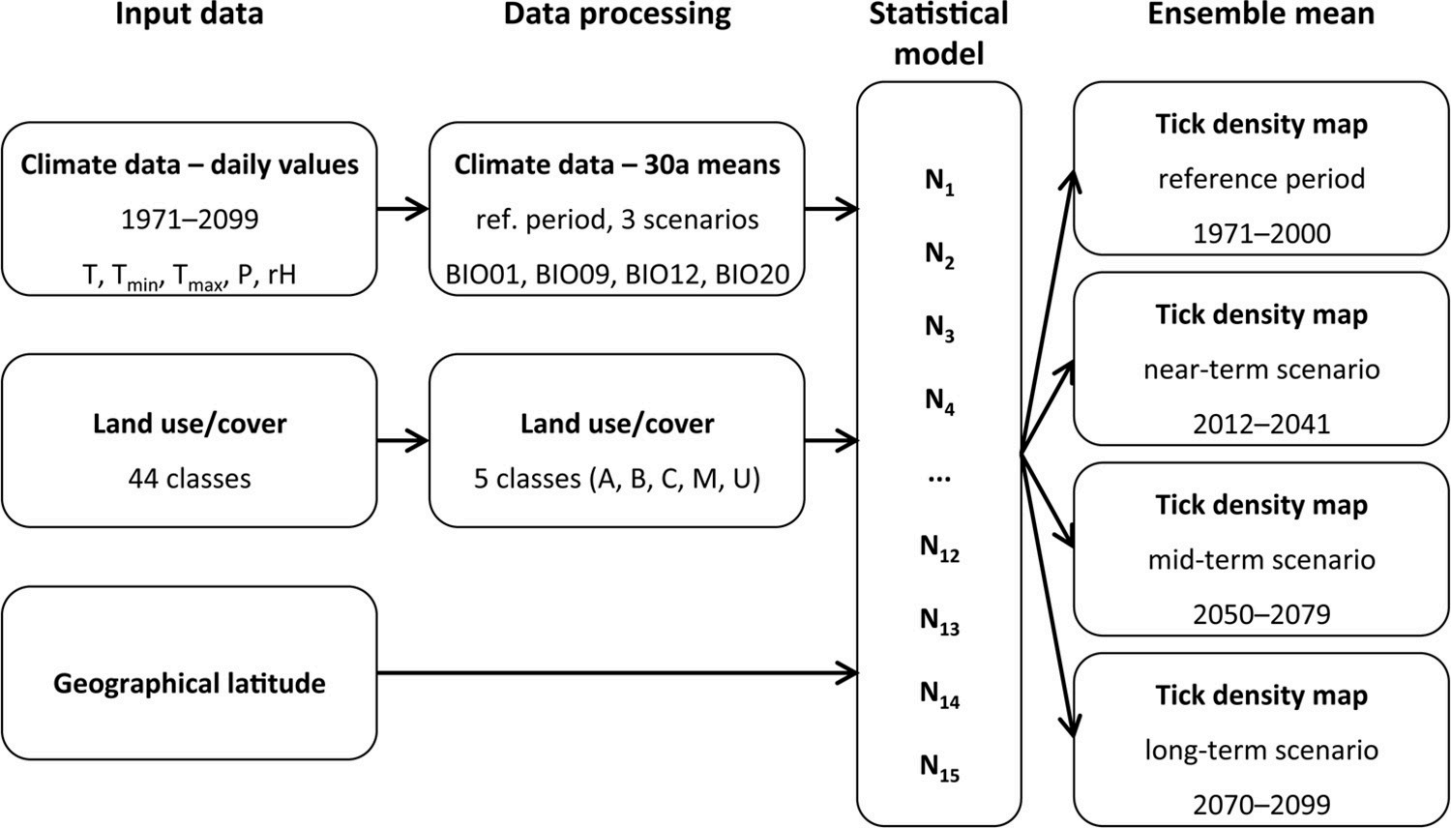
Flowchart of the data processing and implementation of the German-wide tick density model.

As all datasets have different spatial resolutions and map projections, we transformed them to a regular grid with a spatial resolution of 0.2° in longitude and 0.12° in latitude (coordinate reference system WGS84, EPSG:4326) covering the area of 5–16° E and 47–56° N. According to this, the model domain is covered by a 75 x 55 grid with a mean grid cell size of 186 km^2^ (170–201). We applied the technique of ensemble projections to estimate the most likely prediction considering the 15 climate models. We estimated the *I*. *ricinus* nymphal density for each simulation (Fig 4). The 30-years reference period and three 30-years scenarios provides us with the range of the projected future nymphal tick density. Finally, an ensemble mean was calculated for each scenario and anomalies between the scenarios and the reference depicted.

All analyses were conducted using the open-source statistical computing environment R [37]. The package *raster* [38] was used for processing and visualizing the gridded datasets, and the package *dismo* [39] for calculating the bioclimatic parameters.

## Results

### Nymphal activity

#### Nymphal peak activity

**Fig 5:**
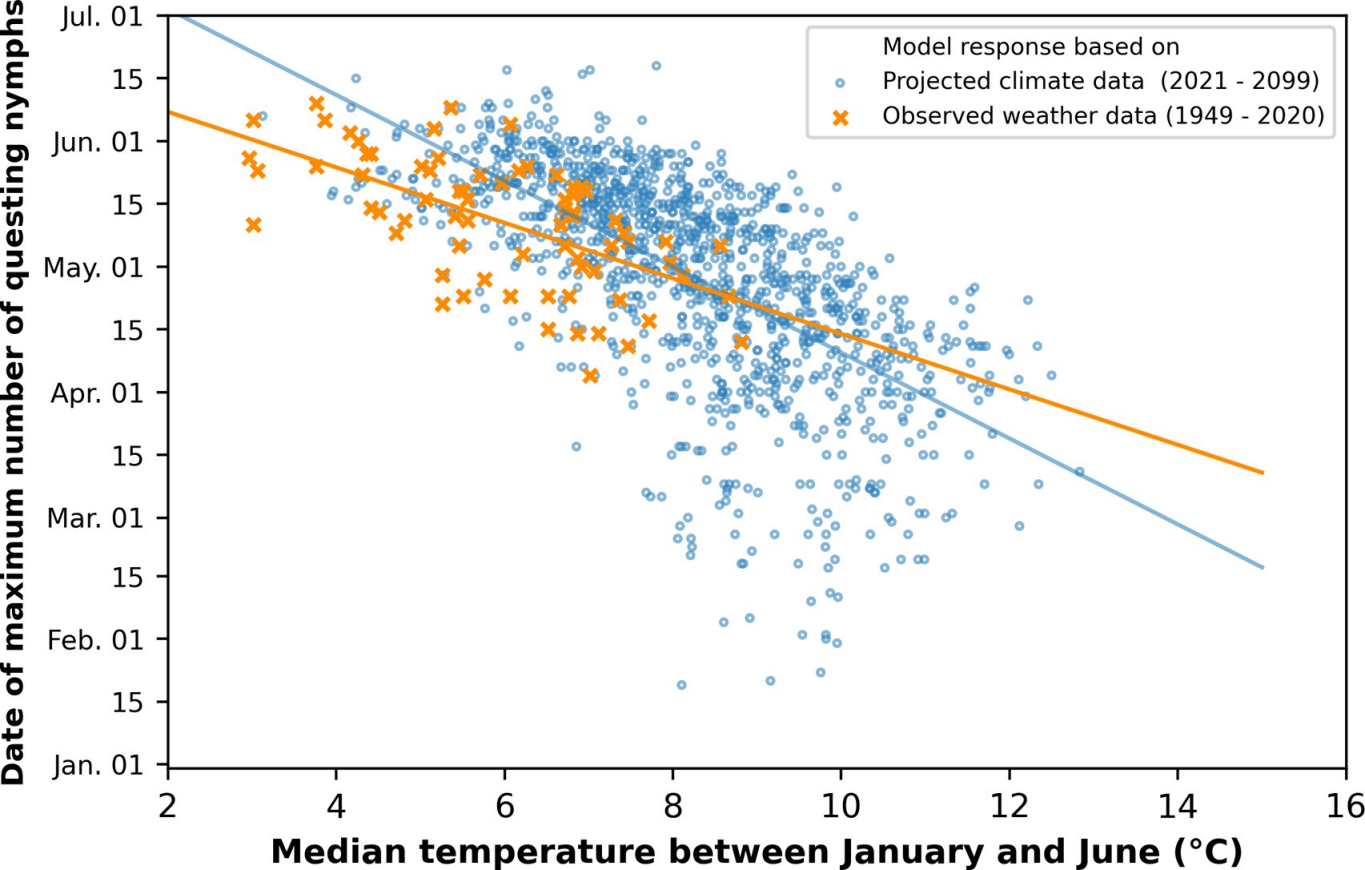
**Relationship of the median temperature between January and June (x-axis) and the date of the maximum number of questing nymphs on the model landscape (y-axis).** Each data point corresponds to a simulation run of a particular year. Orange crosses represent IRIS model runs with observed weather data leading to a correlation coefficient of r = -0.61. Blue circles represent IRIS model runs with projected climate data for the period 2021–2099 leading to a correlation coefficient of r = -0.65. There are 15 data points for each year simulated with data from climate models (blue dots). Each of them represents a different climate model.

We found that the peak activity of questing nymphs shifted from late June to early April as the climate projection data moved further towards into the future, i.e. with increasing median January–June temperature (Fig 5). The spread of simulated peak activities is widened extending from the beginning of June to mid-January. The median January-June temperature of the historical DWD data (1949–2020) lies in the range from 3°C to 9°C (Fig 5, orange crosses). Higher input temperatures are associated with earlier activities peaks in the simulations. The main activity took place less often in June at temperatures from approx. 8.5°C, in May at temperatures from approx. 10°C and finally in mid-April at temperatures from approx. 11°C. For simulations with climate data for the period between 2021 and 2099 (Fig 5, blue circles), the correlation coefficient is r = -0.65. For simulations with weather data from the DWD between 1949 and 2020 it is r = -0.61. It is noticeable that the long-term projection until 2099 fits reasonably well with the trends in observation data up to the year 2020.

#### Most affected season

**Fig 6:**
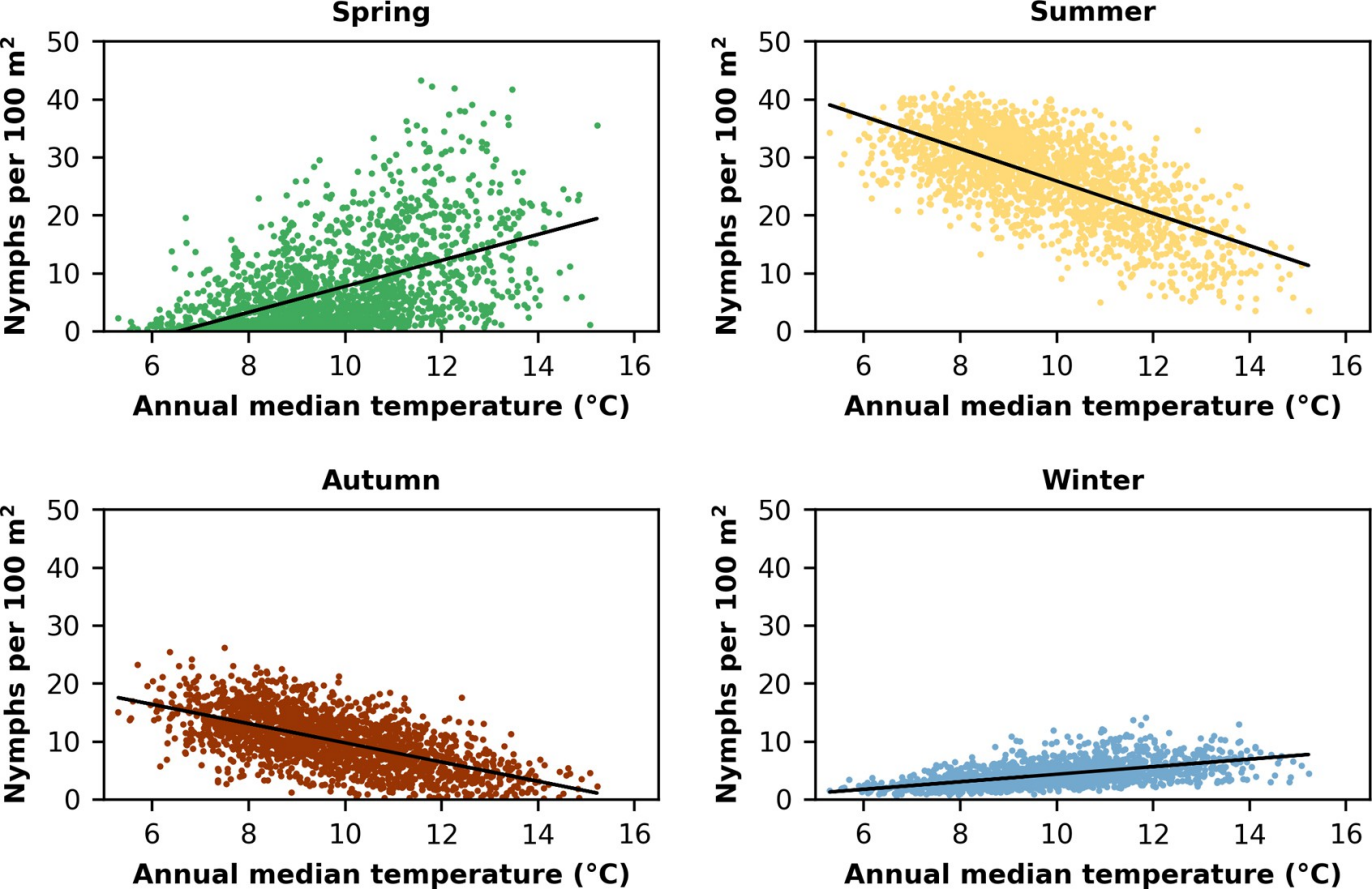
Relationship of the annual median temperature (x-axis) and the density of questing nymphs / 100 m^2^ (y-axis) for the seasons of a year.

Looking at the average density of active nymphs separately for each meteorological season allows us to decompose the above peak shift into distinct components (Fig 6). The warmer the median temperature of the year, we found (a) an increase from low to high activity levels during spring, (b) a decrease from high to low activity levels in summer, (c) a decrease from medium to low activity levels during autumn, and finally (d) a small increase in Winter.

While we found that peak activity moved towards early spring and winter, the effect size of the decreasing activity during summer months is actually 50% larger. Hence, we aimed at capturing this trend when evaluating the minimum activity during summer as proxy for prolonged periods of unfavourable conditions.

#### Minimum questing activity in summer

**Fig 7:**
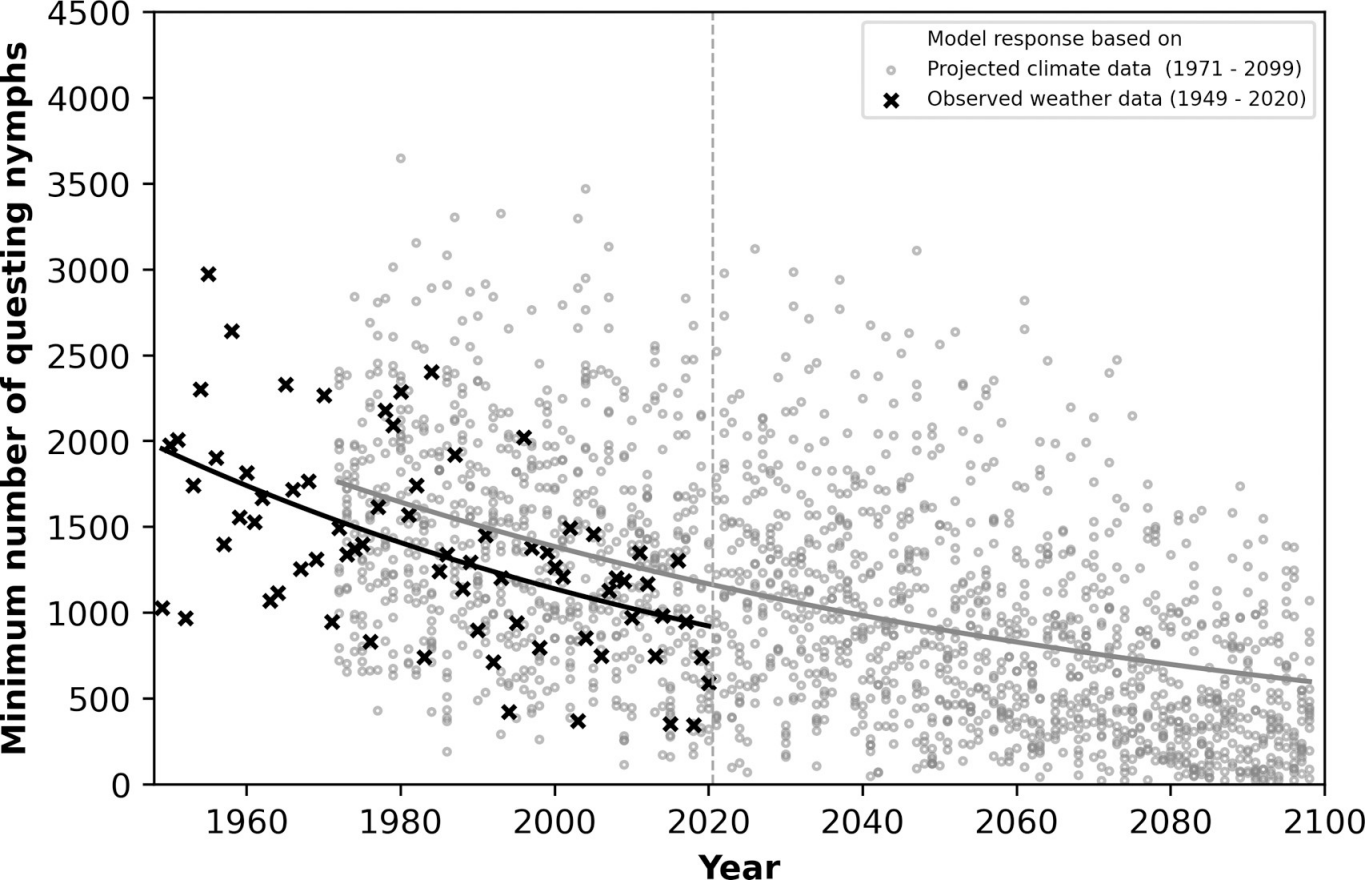
Minimum number of questing nymphs on the simulation landscape in the summer month (June–August) for the period 1949–2099. Simulations were conducted with observed weather data between 1949 and 2020 (black crosses) and with weather data from 15 climate models between 1971 and 2099 (grey circles). The dashed vertical grey line between 2020 and 2021 indicates the border between past and future years.

We found a decreasing trend for the annual minimum questing activity during the summer season (1 June till 31 August) for all years between 1949 and 2099 (Fig 7). The number of active nymphs in the historic period (1949–1971) was on average about 2000 (+- approx. 1000 nymphs). Between 2080 and 2099, it was on average less than 1000 active nymphs. Both sets of simulations, with historic and projected weather data, show large fluctuations between individual years. Interestingly, the climate impact (downward trend) is stronger among the historic climate data (1949–2020) than the trend with the data coming from the climate models.

### Impact of changing climate on the German-wide tick density

**Fig 8:**
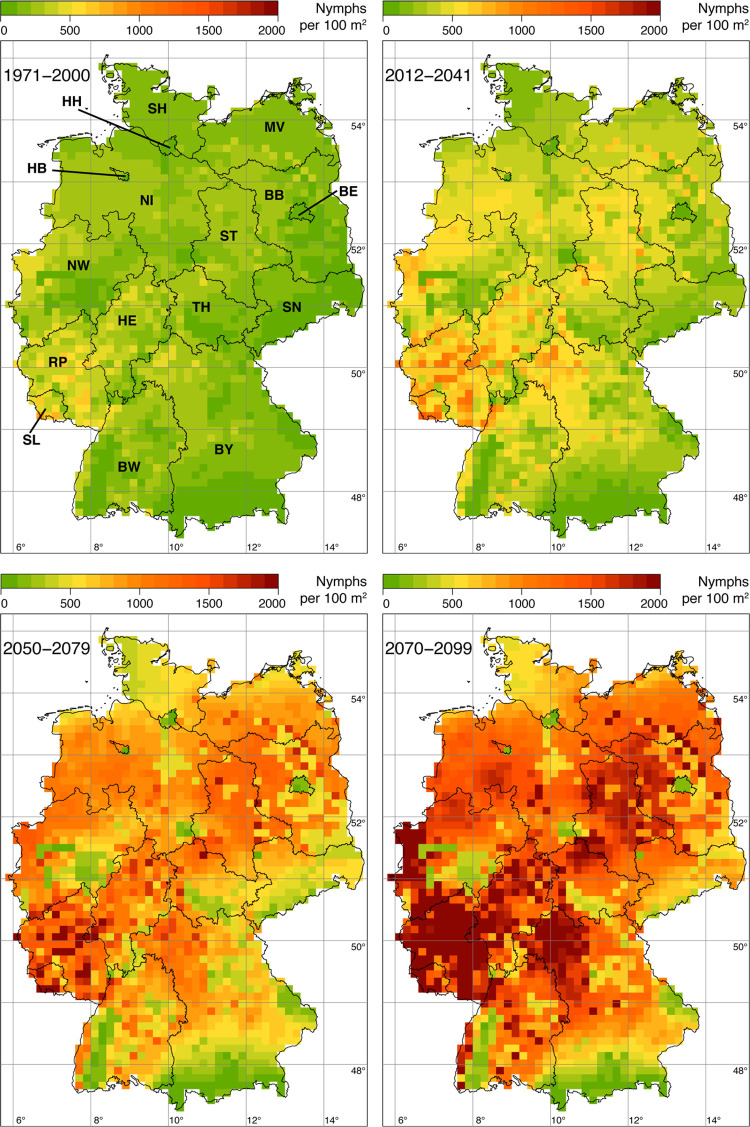
Ensemble means of nymphal *I*. *ricinus* density for the reference period (top left), the near-term scenario (top right), the mid-term scenario (bottom left), and the long-term scenario (bottom right). We set a threshold of 2,000 nymphs / 100 m^2^ which is twice the maximum currently observed annual nymph density in Germany (O. Kahl 2021, pers. comm.). Note that the given density of nymphs / 100 m^2^ should be interpreted as the annual number of *I*. *ricinus* nymphs, which one would collect by monthly flagging an area of 100 m^2^. The abbreviations in the map at the top left indicate the names of the federal states of Germany (BW: Baden-Württemberg, BY: Bavaria, BE: Berlin, BB: Brandenburg, HB: Bremen, HH: Hamburg, HE: Hesse, NI: Lower Saxony, MV: Mecklenburg-Vorpommern, NW: North Rhine-Westphalia, RP: Rhineland-Palatinate, SL: Saarland, SN: Saxony, ST: Saxony-Anhalt, SH: Schleswig-Holstein, TH: Thuringia). State and federal state boundaries were taken from the GADM, the database of global administrative areas (https://gadm.org).

With regard to the spatial impact of changing climatic conditions across Germany under the three scenarios, we found an overall increase of nymphal *I*. *ricinus* densities compared to the reference period. An overview of the simulation results of the model can be found in Table 2. Put simply, as all climatic parameters are increasing, also the nymphal tick densities increases. Fig 8 shows the Germany-wide map of the ensemble means of nymphal tick density for the reference period (1971–2000) and the three climate scenarios. In the reference scenario, we found high nymphal densities in the federal states of Saarland, Rhineland-Palatinate, and in the west of North Rhine-Westphalia. In the short-term scenario (2012–2041), this trend continued towards the north-east (Hesse, Saxony-Anhalt), north (North Rhine-Westphalia, Lower Saxony) and east (Bavaria). In Saarland and Rhineland-Palatinate, tick hotspots with nymphal densities in the range of 1,000 nymphs / 100 m^2^ are already evident. In the mid-term scenario (2050–2079), high tick densities were found nationwide. In large parts of most federal states, densities of >750 nymphs / 100 m^2^ were simulated. Exceptions with relatively low nymphal densities (200 nymphs / 100 m^2^) were found in urban areas of Berlin, Hamburg, Bremen, and parts of the Ruhr area and also in the north of Schleswig-Holstein, the south-west of Baden-Württemberg and in south-eastern Germany (Bavaria, Saxony). The long-term scenario (2070–2099) is qualitatively comparable to the mid-term scenario, but the nymphal densities are noticeably higher. Large contiguous areas with nymph densities of up to 2,000 nymphs / 100 m^2^ can be observed. These hotspots are located in Rhineland-Palatinate, Saarland, in the west of North Rhine-Westphalia, in northern Bavaria and in Saxony-Anhalt.

**Table 2:** Overview of the climatic parameters and the resulting nymphal *I*. *ricinus* densities for the three future scenarios.

Parameter	Near-term scenario (2012–2041)	Mid-term scenario (2050–2079)	Long-term scenario (2070–2099)
Annual mean temperature [°C]	1.5	3.2	4.3
Temperature of the driest quarter [°C]	3.0	7.0	9.5
Annual precipitation [mm/a]	76	135	170
Annual relative humidity [%]	0.3	1.3	0.9
Nymphs per 100 m^2^	176	600	1,028
Gird cells with > 2000 nymphs / 100 m^2^	0 (0.0%)	16 (0.8%)	186 (9.7%)

The values are given as difference to the reference period (1971–2000).

**Fig 9:**
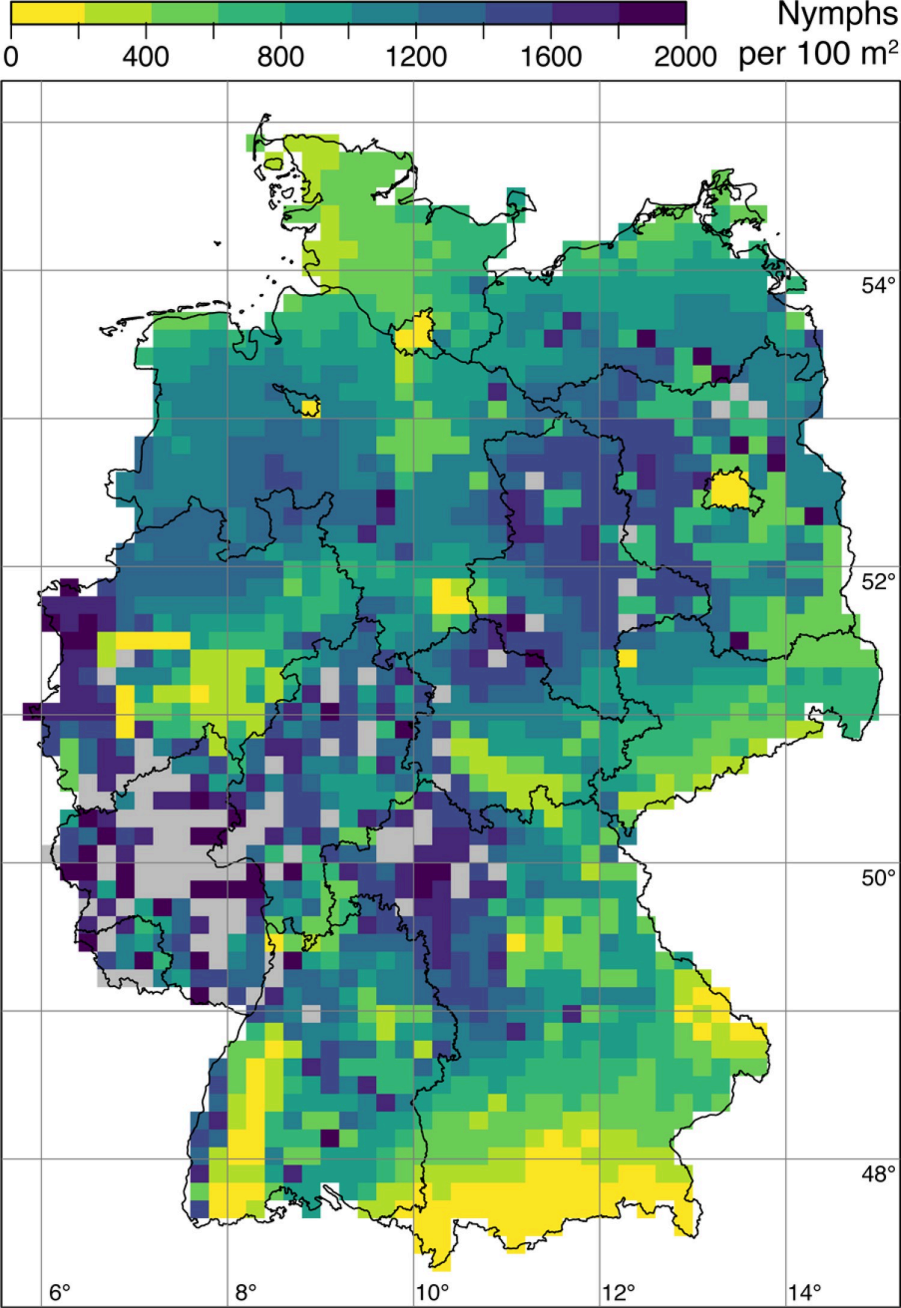
Germany map of nymphal *I*. *ricinus* density differences depicting the change between the reference period (1971–2000) and the long-term scenario (2070–2099). If the density difference exceeds the threshold of 2,000 nymphs / 100 m^2^, the grid cells are indicated in grey. State and federal state boundaries were taken from the GADM, the database of global administrative areas (https://gadm.org).

The Germany-wide density difference map generated using the statistical forecasting model of Brugger et al. [17] shows future hotspots of tick density with up to 2,000 nymphs per 100 m^2^ as well as coldspots without changes of density across Germany (Fig 9). While some regions, especially in the south of the federal states Bavaria, Baden-Württemberg, Saxony, and Thuringia as well as the large cities Berlin, Hannover, and Hamburg show moderate changes in nymph density (coldspots), it increases very strongly in some regions, especially in north-western Brandenburg, Saxony-Anhalt, north-western North Rhine-Westphalia, and northern Bavaria (hotspots). In some regions the density exceeds the threshold of 2,000 nymphs / 100 m^2^, especially in Rhineland-Palatinate, Saarland, Hesse, and northern Bavaria.

## Discussion

The objective of this study was to combine newly available high-resolution climate data with two ecological models. Using these models we derived insights about the future activity and density of *I*. *ricinus* ticks related to climate predictions. For this purpose, we linked time series of climate parameters, i.e. temperature and relative humidity, from recent climate projections to a mechanistic tick population model (IRIS) and a statistical density model.

Looking at the peak nymphal activity versus the median temperature between January and June revealed that increasing temperatures before summer shift the maximum nymphal questing activity towards earlier times of the year. Nymphal tick activation in the model takes place when the daily temperature is within the suitable range. The first half of the year (January–June) was chosen for the year’s temperature characteristics because maximum activity occurred before summer, and later temperatures will not influence earlier tick dynamics. An increase of the before summer months is associated with more days in winter and spring that support tick activity. In general, the observed shifts of peak activity towards earlier times of the year seem plausible and are in accordance with several studies describing it as a result of milder winters [12, 14]. In addition, Gilbert et al. [10] found that *I*. *ricinus* populations could advance their activity season by one month under climate change if only the temperature is considered. Nevertheless, the extent of the observed shift raises questions, for example because in extreme projections the peak nymphal activity occurred even in January. Despite the fact that host seeking winter activity has been observed in the winter of 2006/2007 by Dautel et al. [40], nymphal activity between mid-November and mid-February is considered unusual in central Europe [12].

Secondly, we decomposed this peak activity shift into seasonal components. This observation looks at the shift of maximum peak activity towards earlier times of the year from a different perspective. We found that increasing annual median temperatures result in increased and earlier activity in winter and spring which is in line with Dautel et al. [40]. The effect is more pronounced in spring compared to winter, where lower temperature is still limiting nymph activity. On the other hand, when looking at the summer season, the effect was opposite, i.e. an increasing annual median temperature results in a strong decrease in nymphal tick activity due to heat and drought. Our findings highlight that for the moderately continental climate, characterised by cold winters and warm summers, the associated climate projection would impact tick dynamics mostly in winter and summer. The winter effect was already associated with the earlier start of nymphal activation leading to earlier peaks. The summer effect now is understandable as “dip” from the last of our findings. According to Gray et al. [12], survival and activity of ticks is expected to be reduced in hot summers with low precipitation. Future climate projections predict not only hot and less humid periods for future summer months but also longer continuous periods unsuitable for tick activity. With long-lasting inactivation of ticks, a minimal number of questing ticks is reached, which can be observed in our simulations. That is the reason why the minimum activity of nymphs in summer decreased with advancing years. Indeed, for a specific location in Slovenia, a study by Knap et al. [41] found a correlation between the decrease of questing ticks in summer and the combination of temperature and humidity.

One thing to note about the minimum questing activity in summer is that the decreasing trend is greater in the observed climate (1949–2020) than in the predicted climate (2021–2099). Nevertheless, the general trend of both observed and projected climate was similar. However, after passing the multi-parameter climate forecast through our model, the result indicates that for tick physiology the climate change within the last years might have been already worse than the central tendency of the projected climate range.

With regard to the spatial impact of climate change on the density of *I*. *ricinus* for Germany under the long-term scenario (2070–2099), an unexpected result was that density hotspots with an increase of up to 2,000 nymphs per 100 m^2^ occurred in large parts of Germany. This finding was not anticipated because such high values stand out far from what is considered a high but still biologically plausible density. As mentioned earlier, the current maximum observed annual nymphal density in Germany is approx. 1,000 nymphs / 100 m^2^ (O. Kahl 2021, pers. comm.). Interestingly, there appears to be a spatial association between coldspots where the predicted tick density could change under future climatic conditions and regions where human borreliosis notifications are more frequent today. Conversely, hotspots of predicted tick density change coincided with areas where the notification frequency is low today [42].

Interestingly, the principle trends proposed as a consequence of climate change into the far future could be seen already from observed climate. Although the future trend is extreme and involves large changes and the historic trend is perceived reasonable and changes are considerable, the general patterns predicted with our models already have been realised in reality. This is possibly one reason why it is common to extrapolate the climate response from models based on contemporary knowledge of a system’s behaviour and observations into the distant climatic future (e.g. [21, 43–45]).

In our case, the extrapolation resulted in a temperature-driven shift in the nymphal peak, which led to a projection of ticks questing in winter. It also led to a density estimation using the ecology observed today which resulted in an accumulation of excessive numbers of ticks in future habitats. The implicit assumption of our models and previous applications of climate-only-driven projections of density and activity is that "everything is determined by temperature". This means that all other biotic and abiotic elements of the investigated system either remain equal or are also driven by temperature and relative humidity. For example, Gray et al. [12] ask whether ticks will find enough hosts during the winter as these are then usually less abundant, although it is unclear whether this is also the case in warmer winters. Dobson and Randolph [46] point out that the abundance of vertebrate hosts, specifically deer, is the major biotic determinant of tick population dynamics.

We implicitly assume that tick behaviour observed under today’s climate conditions continues into the future. This treats the organisms as automata that cannot change their functioning although the key signals may alter their meaning. For example, the discussion on the actual drivers of tick behaviour is still ongoing. Some authors argue that ticks are thermometers that start questing when a certain temperature is reached as is stated in the literature (e.g. [3, 12, 14]) and assumed by our model. While others suggest that ticks might also be clocks, i.e. become active based on the sun hours of the day (e.g. [47–49]). This would mean that the temperature range at which their activity is observed today is merely implied by the temperatures usually observed in the relevant parts of the year. More specifically, according to Dautel et al. [40], it has long been assumed that the inactivity of unfed ticks in winter is a behavioural diapause, a genetically determined state that is triggered and controlled by an environmental factor (i.e. in our metaphor the clock) that is neither favourable nor unfavourable in itself, e.g. day length. They found support for the supposition that it is instead a quiescence triggered and terminated by favourable or unfavourable conditions. This would be the thermometer in our metaphor. The IRIS model is primarily temperature dependent. However, if relevant processes are time-dependent, this would have implications for the chosen extrapolation approach. Today, both rule sets would make the same predictions for tick activity but they would diverge completely when extrapolated into the future as the length of day would be unaffected by climate change. This is an illustrative example that it is very important to know whether drivers of activation are time or temperature dependent if we want to extrapolate such a complex system into the future in order to make reliable statements about the influence of climate change on future tick populations.

While day length is unaffected by climate change, the response of ticks could be subject to evolution. But climate change is happening faster than before and is putting high pressure on evolutionary adaptation of many species, with unclear success (e.g. [50–52]). Hence the question arises whether the ticks could adapt without genotypic changes. This kind of adaptation poses a significant issue to ecological modellers: While the laws of nature are generally assumed to be unchanging over space and time [53, 54], the rules governing ecological models are usually based on observations of current responses of the organism which could be contingent, i.e. dependent on the context and history of the specific situation. The dominant behaviours could be completely different when different habitats are considered or when different conditions have shaped the past of an ecosystem. It is therefore not clear whether the rules that we extracted from our survey of the literature would be the same if we repeated that same survey 50 years later. In general, organisms are adaptable and continuously change their behaviour based on environmental conditions, competitive pressure, etc. (e.g. [55]). If ticks behave like organisms that can adapt to changing environmental conditions, and hence also change their behavioural rules, then an extrapolation would be uncertain because it is not clear how they would adapt to future climate and how a rule-based model of their future adapted behaviour would look like. Findings from Gilbert et al. [10] suggest that *I*. *ricinus* has the potential to adapt to changing climates. Also Gray et al. [12] describe *I*. *ricinus* as extremely flexible and adaptable with regard to its seasonal activity making it difficult to determine future scenarios.

To be truly confident in the extrapolation of a rule-based model, we would need to include all possibilities for adaptation of a given organism in the rule set itself. Even leaving aside whether such descriptions could be supported by empirical data on a species’ evolutionary past (e.g. through fossil records) or by considering geographically distant populations, this would eventually devolve into a “theory of everything”, a “world model”, which would need to encompass a description of evolution itself and have very little explanatory power for any concrete ecosystem.

Similar to how rule-based models can be driven outside of the range in which their rules apply, also linear models, such as the presented model by Brugger et al. [17] still provide answers even if the parameter range of an approximately linear response has been left. Our results have shown that biologically plausible nymphal densities were sometimes significantly exceeded. Therefore, the results of this modelling approach must also be interpreted with caution if one wants to use it to extrapolate a complex systems into the distant future.

Finally, the question arises whether purely predictive models such as Machine Learning could be sufficient to reduce the future risk of tick contact. However, in contrast to mechanistic models, purely predictive models are unable to explain the relevant processes, e.g. of tick activation (e.g. [56]). If the goal is to merely avoid the contact with ticks on risk days and in risk habitats, these types of models may be sufficient for passive adaptation to future changes. For example on days with predicted high densities, people could avoid the identified risk areas. But if the greater goal is to actively adapt, e.g. by changing land management strategies such as the vaccination of certain host species to mitigate the increase in ticks, this would require explanatory models to identify the relevant processes and impacts where mitigation measures can be implemented.

In summary, it can be stated that the presented model analyses suggest extreme changes of tick dynamics and density with pronounced shift of maximum peak activity towards earlier times of the year and dramatic increase of tick density across Germany. Our results are generally consistent with trends in the current literature on the topic, but were in some cases much more extreme (nymphal peak in January, density change of 2,000 nymphs / 100 m^2^). Therefore, we conclude that caution is useful when extrapolating climate data-driven models into the distant future and only climate data drives the model projection.

## Supporting information

S1 Appendix IRIS model description following the ODD protocol. (DOCX)

S2 Appendix Results of the IRIS model calibration. (DOCX)

S3 Appendix Derived quantities contrasted with the annual mean temperature, the annual median temperature and years on the x-axis. (DOCX)
