# Supplementary material for: Model-based extrapolation of ecological systems under future climate scenarios: The example of Ixodes ricinus ticks
Source: PLoS One. 2022 Apr 22;17(4):e0267196. doi: 10.1371/journal.pone.0267196 (PMC9032420; doi:10.1371/journal.pone.0267196)
Supplement: S1 Appendix — (DOCX) [file pone.0267196.s001.docx]

**ODD Protocol**

This model description follows the Overview, Design concepts and Details (ODD) protocol. ODD is a standard format to systematically describe models in a comprehensive and transparent way aiming to allow for model replication (Grimm et al., 2006; Grimm et al., 2010, Grimm et al., 2020).

In the following, we describe the model IRIS (Ixodes RIcinus Simulator). It is a cohort-based and spatially-explicit population model to simulate local dynamics of *Ixodes ricinus* ticks under climate change. It consists of behavioural rules of ticks and takes weather and climate data as input to simulate the seasonal spatio-temporal abundance and questing activity of ticks of different life cycle stages over a year with daily time steps. The model dynamic is driven by the population response to prevailing temperatures and relative humidity which are modulated by the spatial distribution of habitat types.

1. **Purpose**

The overall purpose of the IRIS model is to understand the impact of climate change on the spatial and temporal abundance of *I. ricinus* ticks on the local scale. As ticks are highly relevant vectors of Lyme borreliosis, it is important to gain a better understanding of their future climate-dependent dynamics to assess the exposure risk for humans.

1. **Entities, state variables and scales**

**2.1 Entities**

The entities of the model are *I. ricinus* ticks that are tracked as cohorts. Each cohort represents a tick life cycle stage together with a behavioural state. Three tick life cycle stages are possible: (1) *larvae*, (2) *nymphs* and (3) *adults*. Each life cycle stage can adopt one of four behavioural states: (i) *questing*, (ii) *inactive*, (iii) *engorged* and (iv) *late engorged*. The latter is a special case of the behavioural state *engorged* which applies only to larvae and nymphs (see Section 7.5 for details). Hence, in total there are 11 tick cohorts in the model. These cohorts are present in every grid cell of the model landscape, e.g. active nymphs in grid cell (0, 0) belong to a different cohort than active nymphs in grid cell (3, 5). For model evaluations the cohorts are considered in total.

Furthermore, the model is composed of grid cells that form a landscape and serve as habitat. They can be localised based on their x and y coordinates. In addition to the tick cohorts, each grid cell is assigned a habitat type and weather characteristics, i.e. temperature and relative humidity. Three idealised habitat types exist in the model: (a) *forest*, (b) *ecotone* and (c) *meadow*. These factors then determine the behaviour and number of ticks in a particular grid cell. A visualisation of the model landscape with habitat types and tick cohorts is displayed in Fig 1.


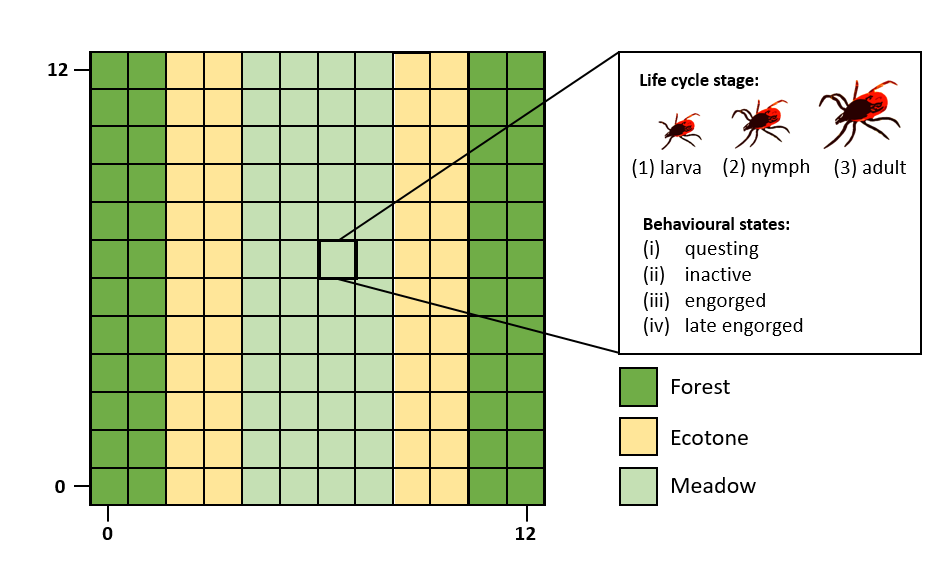


**Fig 1: Schematic representation of the IRIS model landscape.** It consists of 12 x 12 grid cells with periodic boundary conditions. The colour of each grid cell indicates the habitat type: forest (dark green), ecotone (light brown) and meadow (light green). Each cell contains tick cohorts consisting of one of three life cycle stages (larva, nymph, adult) together with one of four behavioural states (questing, inactive, engorged and late engorged).

**2.2. State variables**

By implementing IRIS using an Entity Component System (ECS) framework, the state variables of the model are organised in so-called ECS components (see Section 4 for details). Table 1 provides an overview of the state variables of the IRIS model.

**Table 1: Overview of state variables of the IRIS model.**

| **Component** | **Variable** | **Type** | **Possible Values** | **Description** |
| --- | --- | --- | --- | --- |
| Time Step | current | int | 1,...,365 | Current time step |
| Randomness | rng | RandomGenerator | - | Pseudo Random Number Generator |
| Position | x | int | 1,...,12 | x coordinate |
|  | y | int | 1,...,12 | y coordinate |
| Habitat | FOREST | Type | - | Habitat type forest |
|  | ECOTONE | Type | - | Habitat type ecotone |
|  | MEADOW | Type | - | Habitat type meadow |
| TickAbundance | LARVAE_QUESTING | int | N | No. of questing larve |
|  | NYMPHS_QUESTING | int | N | No of questing nymphs |
|  | ADULTS_QUESTING | int | N | No. of questing adults |
|  | LARVAE_INACTIVE | int | N | No. of inactive larvae |
|  | NYMPHS_INACTIVE | int | N | No. of inactive nymphs |
|  | ADULTS_INACTIVE | int | N | No. of inactive adults |
|  | LARVAE_ENGORGED | int | N | No. of engorged larvae |
|  | NYMPHS_ENGORGED | int | N | No. of engorged nymphs |
|  | ADULTS_ENGORGED | int | N | No. of engorged adults |
|  | LARVAE_LATE_ENGORGED | int | N | No. of late engorged larvae |
|  | NYMPHS_LATE_ENGORGED | int | N | No. of late engorged nymphs |
| Temperature | meanTemperature | double | R | Mean temperature |
|  | minTemperature | double | R | Minimum temperature |
|  | maxTemperature | double | R | Maximum temperature |
| Humidity | humidity | double | 0,...,100 | Relative humidity |

In addition, each grid cell is provided with a unique identification number used for internal processing by the ECS framework. This entityID is linked to the component Position within the ECS resource SpatialIndex.

**2.3. Scales**

The spatial scale of the model corresponds to an area of 120 m x 120 m. The model landscape consist of 12 x 12 = 144 grid cells. A grid cell has an area of 10 m x 10 m = 100 m^2^. The reason for this grid cell size is that tick densities are usually expressed in numbers per 100 m^2^ (see e.g. Table 2 in Boehnke et al., 2015). This is because the standard method of collecting host-seeking ticks (called flagging) is typically done over a sampling area of 100 m^2^ (Schulz et al., 2014; Brugger et al., 2016).

A single simulation run of IRIS covers a period of one year with daily time steps. This time scale captures the local population dynamics of ticks quite well and is the most appropriate for the model purpose. A smaller time scale, e.g. with hourly time steps, would make little sense as we are looking at averaged behaviour of tick cohorts rather than exact behaviour of individual ticks. A larger time scale, e.g. with weekly or monthly time steps, would also not make much sense as many biological processes happen during a day such as the decision of a tick to quest or the attachment to a host and thus the translocation to another grid cell. Furthermore, required input data for temperature and relative humidity is available on a daily basis from German weather stations and climate simulation models (see Section 6 for details). In terms of runtime daily time steps are also feasible.

1. **Process overview and scheduling**

The IRIS model simulates one year from January 1 to December 31 and is executed in daily discrete time steps. A leap year also includes 29 February. In each time step, the following model processes are executed for every grid cell and the state-variables are updated in the following order:

1. Weather
2. Activity
3. Feeding
4. Development
5. Freezing
6. Desiccation

The process Weather is responsible for setting the daily temperature and relative humidity and updates the state variables *meanTemperature*, *minTemperature*, maxTemperature and *relativeHumidity*. The process Activity involves the active search for a host (also known as *questing*) or putting ticks into a state of inactivity. The process Feeding controls the feeding success of ticks, i.e. the successful attachment of a ticks to a hosts. Furthermore, it includes a translocation process by which ticks are moved to other locations on the model landscape, i.e. moved to a cohort in another grid cell. The process Development involves the transition from one life cycle stage to the next. The processes Freezing and Desiccation control tick mortality in case of unfavourable environmental conditions. All processes (except for Weather) update the state variable stage of each grid cell. Each process is described in more detail in Section 7. A visualisation of the state transitions of ticks through the model processes is displayed in Fig 2.


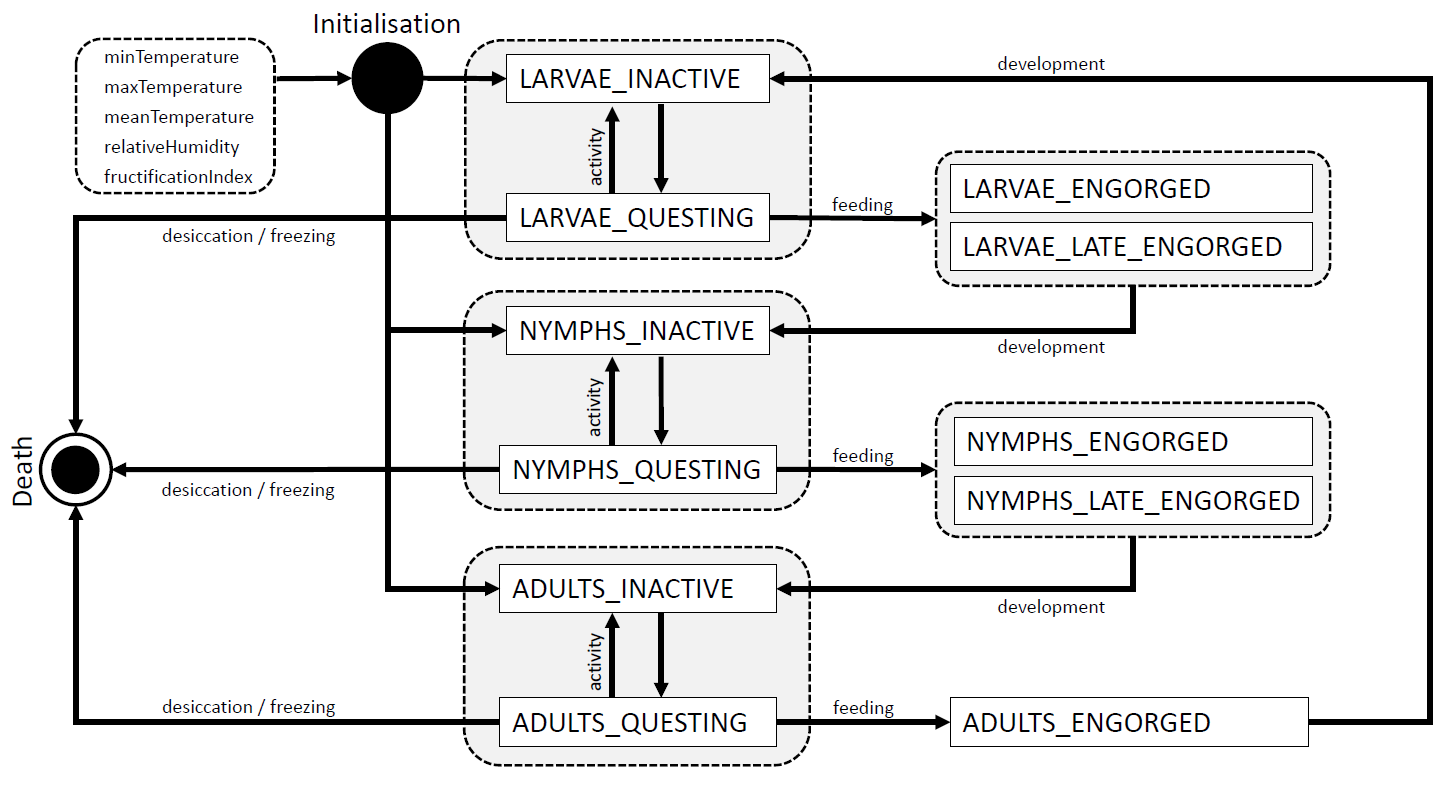


**Fig 2: State transition diagram of tick cohorts.** The 11 cohorts are represented by white boxes and the transitions between the cohorts are modelled by black arrows. The labelling on the arrows indicates which method of a process is responsible for the respective transition. Ticks that desiccate or freeze are transitioned to the end state "death" (indicated by the black dot with black circle) and thus removed from the system. The initial state "Initialisation" (marked by the black dot) indicates the cohorts that are initialised at the beginning of a simulation. All cohorts can be initialised individually. For the sake of clarity, the diagram only shows the default case with inactive larvae, nymphs or adults being initialised.

1. **Design Concepts**

**Basic principles:** IRIS was implemented in Java using version 2.3.0 of the Artemis-odb Entity-Component-System (ECS) framework.

The model landscape is modelled with periodic boundary conditions to prevent a model artefact where ticks are concentrated in the centre. This means that the neighbours of the grid cells at the edge of the two-dimensional landscape projection are the grid cells on the opposite side. Daily temperature and relative humidity influence the behaviour of the modelled ticks and thereby drive the model dynamics. With the input of future weather data from climate simulations, the model is able to simulate the expected future abundance of ticks.

The general way to model state transitions between each two tick cohorts is to determine the number of individuals that are subtracted from one cohort and added to another cohort using a probability rate and stochastic rounding (see also part "Stochasticity" below). This is illustrated by the following pseudo code with two fictitious cohorts A and B:

newTicksCohortB = roundRandom(getNumberTicksCohortA() * probabilityRate);

addTicksCohortB(NumberTicksCohortB + newTicksCohortB);

addTicksCohortA(NumberTicksCohortA - newTicksCohortB);

**Stochasticity:** Stochasticity is incorporated into the model in two ways. First, stochastic rounding is used to break down probability rates to the individual tick level because state transitions between tick cohorts are based on rates, but integer numbers of individuals are stored into the cohorts. Otherwise, the number of ticks in a cohort could never become zero in some cases when normal rounding was applied. A seedable random number generator is used to determine random numbers. Secondly, the translocation process of the submodel *Feeding* described in Section 7.5 uses stochasticity through distance-dependent probabilities. The translocation to a distant grid cell occurs with a lower probability than the translocation to a nearby grid cell.

**Emergence:** The main output of the model are numbers of *I. ricinus* ticks in each of the 11 cohorts. The numbers in each cohort of each grid cell emerge from the interaction of environmental conditions, i.e. the temperature and relative humidity, the habitat type and the behavioural rules of ticks.

**Adaptation:** The adaptive behaviour of ticks consists in their reaction to daily temperature and relative humidity to avoid desiccation or freezing or to seek for a host. When certain temperature and humidity values fall below or exceed certain thresholds in their respective habitats, ticks switch between their behavioural states *questing* and *inactive*. Since the model is cohort-based, adaptive behaviour does not take place at the level of an individual tick, but is aggregated at the level of the respective cohort. A share of the number of ticks in a given cohort is shifted to the other cohort in response to environmental influences. The adaptive behaviour is described in detail in Section 7.

**Sensing:** Ticks are assumed to sense temperature and relative humidity. Due to the cohort-based nature of the model, sensing does not take place at the level of an individual tick but instead at the level of the respective cohorts.

**Collectives:** Since this model is cohort-based, all of the eleven model cohorts can be interpreted as collectives that combine a sub-population of a certain number of *I. ricinus* ticks in each grid cell. Interaction between cohorts of different grid cells take place via the translocation process implemented in the Feeding component (see Section 7.5 for details).

1. **Initialisation**

The initialisation of the model involves the following steps. First, IRIS is started by calling the main method of one of the classes in the project folder `experiments`. The class *AdHoc* can be called to simulate a single year. It is also possible to pass individual simulation parameters to the *AdHoc* class. The other two classes (*SensitivityAnalysis*, *SensitivityAnalysisLN*) in the `experiments' folder are used to carry out sensitivity analyses. The simulation parameters are already defined there within these classes. When calling the main method of the class *AdHoc* individual command line parameters, if available, are first passed to the model (Table 2). These parameters are used to call the method *run()* in the class *Model*.

The cohorts are initialised with a certain number of ticks assuming that these initial values are the result of the tick life cycle of the previous year. This way the multi-year life cycle of the modelled ticks is captured indirectly. In principle, all of the 11 cohorts present in the model can be initialised individually. By default only the cohorts with inactive ticks, i.e. LARVAE_INACTIVE, NYMPHS_INACTIVE and ADULTS_INACTIVE are initialised. With this, we assume that most ticks are inactive on 1 January.

**Table 2: Overview of model input parameters.**

| **Parameter** | **Default value** | **Type** | **Description** |
| --- | --- | --- | --- |
| seed | 42 | int | random seed |
| weather | - | String | path to weather input file |
| output | - | String | path to set directory of output files |
| initialLarvae | 150 | int | initial number of inactive larvae |
| initialNymphs | 150 | int | initial number of inactive nymphs |
| initialAdults | 150 | int | initial number of inactive adults |
| activationRate | 0.022 | float | activation rate |
| summary | true | boolean | write only summary outputs |

Afterwards, the random number generator is initialised with a random seed and the world configuration of the Entity Component System is assembled. This includes the registration of the ECS Systems *Activity*, *Feeding*, *TickLifeCycle*, *Weather* and the ECS resources *Parameters*, *Randomness*, *SpatialIndex* and *TimeStep*. This is followed by the initialisation of the model landscape, i.e. each grid cell is assigned a position (*x, y*) and a habitat type (forest, ecotone, meadow). In addition, the ECS components Habitat, Humidity, Position, Temperature and TickAbundance are added to the grid cells (i.e. the ECS entities).

**5.1 Adjusting the initial number of larvae using beech mast fructification data**

If data is available the model is able to use beech mast fructification data of the European beech *Fagus sylvatica* to adjust the initial number of larvae at the beginning of a simulation run. The reason for this adjustment can be explained as follows: Small rodents serve as host animals especially for *I. ricinus* larvae (Cayol et al., 2017). A higher density of small rodents in a given year causes an increase in the population of larvae and nymphs in the following year (Brugger et al., 2018). Data on beech mast can be used to estimate rodent populations, because a year with high mast production leads to an increase in the rodent population in the following year due to higher food supply (Clement et al., 2009). Hence, we assume that the initial number of larvae at the beginning of a simulation run of a given year is influenced by the beech mast two years before.

The type of beech mast is divided into four classes and given as an index. Based on this fructification index, the model adjusts the initial number of larvae at the beginning of a simulation run. The default number of initial larvae is reduced if when the second year before the simulated year is not a mast year with full fructification. The strength of the reduction depends on the type of beech mast, i.e. the fructification index. Table 3 gives an overview over the adjustment values. Information on the beech mast input data can be found in Section 6.

**Table 3: Overview of adjustment values to adjust the initial number of larvae based on the fructification index.**

| **Fructification index** | **Description** | **Adjustment value** | **Reference** |
| --- | --- | --- | --- |
| 1 | absent fructification | 0.25 | own estimation |
| 2 | scarce fructification | 0.5 | own estimation |
| 3 | common fructification | 0.75 | own estimation |
| 4 | full fructification | 1.0 | own estimation |

1. **Input data**

IRIS uses observed weather and simulated climate data and optionally data on beech mast as input data. In detail, the model requires the following input data on a daily basis (see Table 5 for a more detailed overview of the input parameters):

- Daily mean near-surface air temperature [°C]
- Daily Maximum Near-Surface Air Temperature [°C]
- Daily Maximum Near-Surface Air Temperature [°C]
- Daily near surface relative humidity [%]

**6.1 Observed weather data**

Observed daily temperature and humidity data was included in the model both for model calibration and simulation of past years for the period between 1949 and 2020 from the German Weather Service (2021). In particular, the following data was used from the weather station in Regensburg (Station ID: 4104; Location: 12.1023° E, 49.0426° N):

- Daily mean of temperature at 2 m height (TMK, [°C])
- Daily minimum of air temperature at 2 m height (TNK, [°C])
- Daily maximum of air temperature at 2 m height (TXK, [°C])
- Daily mean of relative humidity (UPM, [%])

Since the weather station in Regensburg is located at an altitude of 365 metres, but the sampling site in Haselmühl for which the model calibration and simulations were carried out is located at a higher altitude of 430 metres, the temperature values from the DWD data set were adjusted by according to the dry-adiabatic temperature gradient. Specifically, the values were reduced by -0.64 = (430 - 365) / 100 x -0.98 °C. This adjustment must always be made individually for each location for which simulations are to be carried out whenever its altitude differs substantially from the altitude of the location of the measurement of temperatures.

**6.2 Climate simulation data**

Climate simulation data covering Germany with a resolution of 0.11° x 0.11° (~12.5 km) was provided by The Climate Service Center Germany (GERICS). A total of 15 combinations of Global Change Models (GCMs) and Regional Chance Models (RCMs) were provided for the RCP8.5 scenario, i.e. the scenario that exceeds 4K global warming (see Table 4 for an overview). Bias-adjusted EURO-CORDEX simulations (Jacob et al., 2014) were provided for the following parameters:

- Bias Adjusted Near-Surface Air Temperature (tas, [K])
- Bias Adjusted Daily Maximum Near-Surface Air Temperature (tasmax, [K])
- Bias Adjusted Daily Minimum Near-Surface Air Temperature (tasmin, [K])

Bias adjustment is a post-processing method to identify and adjust for possible biases between observed and simulated climate variables of the RCM output (Teutschbein et al., 2012; Maraun et al., 2016). In addition, homogenised EURO-CORDEX simulations were provided for the following parameter:

- Near-Surface Relative Humidity (hurs, [%])

Homogenisation means that non-climatic factors such as relocation of weather stations or changing of measuring instruments are detected and removed from the climate data to improve the overall quality (Costa et al., 2009; Ribeiro et al., 2016).

Table 4: Overview of EURO-CORDEX simulations used as model input. For the 15 GCM-RCM combinations the mean annual temperature (BIO01) in Germany for four periods are given. All simulations were run with the RCP8.5 scenario with the r1i1p1 driving ensemble, only the two MPI-M models were run with the r3i1p1 ensemble.

| **Institute** | **Driving global climate model (GCM)** | **Downscaling regional climate model (RCM)** | **Reference period**  **1971–2000** | **Near-term scenario**  **(1.5 K)**  **2012-2041** | **Mid-term scenario**  **(3.0 K)**  **2050–2079** | **Long-term scenario**  **(4.0 K)**  **2070-2099** |
| --- | --- | --- | --- | --- | --- | --- |
| Canadian Centre for Climate Modelling and Analysis (CCCma) | CanESM2 | CCLM4-8-17 (v1) | 8.6 ± 0.7 | 9.8 ± 0.7 | 11.9 ± 1.0 | 13.2 ± 0.9 |
|  |  | REMO2015 (v1) | 8.6 ± 0.7 | 9.7 ± 0.7 | 11.4 ± 0.9 | 12.4 ± 0.9 |
| Institute Pierre Simon Laplace (IPSL) | IPSL-CM5A-MR | WRF381P (v1) | 8.6 ± 0.7 | 10.1 ± 0.6 | 11.7 ± 0.8 | 12.5 ± 0.6 |
|  |  | RACMO22E (v1) | 8.6 ± 0.9 | 9.9 ± 0.6 | 11.8 ± 0.8 | 12.6 ± 0.7 |
|  |  | RCA4 (v1) | 8.6 ± 1.0 | 9.8 ± 0.5 | 11.6 ± 0.8 | 12.4 ± 0.8 |
| Met Office Hadley Centre (MOHC) | HadGEM2 ES | ALADIN63 (v1) | 8.6 ± 0.9 | 10.4 ± 0.9 | 12.1 ± 0.9 | 13.4 ± 1.0 |
|  |  | HIRHAM5 (v2) | 8.6 ± 0.7 | 10.3 ± 0.8 | 12.1 ± 0.9 | 13.3 ± 0.9 |
|  |  | REMO2015 (v1) | 8.6 ± 0.9 | 10.3 ± 0.9 | 11.9 ± 0.9 | 13.4 ± 1.1 |
|  |  | RegCM4-6 (v1) | 8.6 ± 0.8 | 10.2 ± 0.8 | 12.1 ± 0.8 | 13.3 ± 0.8 |
|  |  | WRF381P (v1) | 8.6 ± 0.8 | 10.1 ± 1.1 | 11.7 ± 1.0 | 12.7 ± 0.9 |
|  |  | RACMO22E (v2) | 8.6 ± 0.9 | 10.3 ± 0.8 | 12.3 ± 0.9 | 13.5 ± 0.9 |
|  |  | HadREM3-GA7-05 (v1) | 8.6 ± 0.8 | 10.6 ± 0.8 | 12.6 ± 1.0 | 13.9 ± 1.0 |
|  |  | RCA4 (v1) | 8.6 ± 0.9 | 10.2 ± 0.9 | 11.8 ± 0.9 | 13.1 ± 1.0 |
| Max-Planck-Institute for Meteorology (MPI-M) | ESM-LR | REMO2015 (v1) | 8.6 ± 0.7 | 10.0 ± 0.5 | 10.9 ± 0.8 | 11.9 ± 0.7 |
|  |  | RCA4 (v1) | 8.6 ± 0.6 | 9.8 ± 0.6 | 11.1 ± 0.8 | 12.1 ± 0.7 |

The climate data was provided in NetCDF format. The weather time series of a single location were extracted using a script and saved in a *.csv file. During this process, the temperature variables *tas*, *tasmax* and *tasmin* were converted from Kelvin to Celsius (°C = K - 273.15).

**6.3 Beech mast fructification data**

IRIS is able to use beech mast fructification data of the European beech *Fagus sylvatica*. This data can be used to adjust the initial number of larvae (for details see Section 5.1). Beech mast fructification data is given as an annual index. For the validation of the model we have used the values from (Brugger et al., 2018). A larger data set with fructification data between 1954 an 2016 is available from (Konnert et al., 2016).

**Table 5: Overview of model input data.** The table shows the input variables that are processed by the model. These include the daily weather data, which can come from weather stations or climate simulations and the beech fructification index, whose values come from Konnert et al. (2016).

| **Parameter** | **Symbol** | **Type** | **Description** |
| --- | --- | --- | --- |
| meanTemperature | *t_mean* | double | Daily mean temperature |
| minTemperature | *t_min* | double | Daily min temperature |
| maxTemperature | *t_max* | double | Daily max temperature |
| humidity | *h* | double | Daily humidity |
| fructification | *f* | int | Fructification index |

1. **Submodels**

**7.1 Development**

This sub model is responsible for the development from one tick life cycle stage to the next. It is part of the TickLifeCycle component and implemented in the method development(). The life cycle of *I. ricinus* ticks consists of four life cycle stages: eggs, larvae, nymphs, adults. Once the eggs have hatched into larvae, each further life cycle stage requires a blood meal to develop to the next life cycle stage (Gray, 1991). IRIS models the life cycle stages larvae, nymphs and adults.

The development from one life cycle stage to the next takes place over specific periods of time. The development period begins at the beginning of July and ends either in early or mid-October, depending on the life cycle stage (O. Kahl 2020, pers. comm.). Since only engorged ticks can develop to the next life cycle stage, their number is subtracted from the respective cohort with behavioural state *engorged* and added to the cohort of the next life cycle stage with behavioural state *inactive*. We assume that all engorged ticks will have developed at the end of the development period. The values of the start and end times of the development period are implemented as constants (see Table 6). The oviposition of adult ticks is not modelled explicitly. It is assumed that the number of new larvae depends on the number of engorged adult ticks.

**Table 6: Overview of start and end times of *I. ricinus* developmental phases.** The second column contain the values of the time step at which the development from one life cycle stage to the next starts or ends.

| **Time period** | **Time step** | **Parameter Name** | **Type** | **Reference** |
| --- | --- | --- | --- | --- |
| Early July | 181 | BEGIN_OF_DEVELOPMENT | int | O. Kahl 2020, pers. comm. |
| Mid-October | 289 | END_OF_DEVELOPMENT_LARVAE_TO_NYMPHS | int | O. Kahl 2020, pers. comm. |
| Early October | 274 | END_OF_DEVELOPMENT_NYMPHS_TO_ADULTS | int | O. Kahl 2020, pers. comm. |
| Mid-October | 289 | END_OF_DEVELOPMENT_ADULTS_TO_LARVAE | int | O. Kahl 2020, pers. comm. |

**7.2 Desiccation**

This sub model controls the desiccation of ticks. It is implemented in the method *desiccation()* in the class *TickLifeCycle*. In general ticks need at least 80% relative humidity in their local environment to survive (Medlock et al., 2013; Gray et al., 2016; Hauser et al., 2018). When the relative humidity falls below this level off-host ticks start to dry out and will eventually die unless the relative humidity rises again. Survival at lower relative humidity is possible but also requires lower temperatures (Ostfeld and Brunner, 2015). Due to the favourable microclimatic conditions, ticks prefer woodland habitats and have a higher abundance there than for example in meadows (Lindstrom and Jaenson, 2003; Boehnke et al., 2015).

In each time step this sub model checks the relative humidity and temperature. If these exceed certain thresholds a proportion of all questing ticks that are in such a grid cell will desiccate (see Table 7). The exact desiccation rate depends on the habitat type of the grid cell. Thereby we capture the favourable effect of the more humid microclimate within a forest compared to a meadow on tick survival. Desiccated ticks are removed from the modelled population.

**Table 7: Overview of habitat dependent desiccation rates and threshold values.**

| **Parameter** | **Habitat** | **Value** | **Unit** | **Reference** |
| --- | --- | --- | --- | --- |
| DESICCATION_MINIMAL_HUMIDITY | - | 80.0 | % | Medlock et al., 2013 |
|  |  |  |  | Gray et al., 2016 |
|  |  |  |  | Hauser et al., 2018 |
| DESICCATION_MINIMAL_MEAN_TEMP | - | 15.0 | °C | Ostfeld & Brunner, 2015 |
| DESICCATION_RATE | Forest | 0.02 | 1 / day | own estimation |
|  | Ecotone | 0.05 | 1 / day | own estimation |
|  | Meadow | 0.10 | 1 / day | own estimation |

**7.3 Freezing**

The sub model *Freezing* is responsible for modelling the effect of extreme cold on ticks. It is part of the ECS component *TickLifeCycle* and implemented in the method *freezing()*. *I. ricinus* ticks are quite resistant to low temperatures (Gray et al., 2009). But they can die when exposed to extreme cold with temperatures lower than approx. minus 15 °C (Ostfeld and Brunner, 2015). Especially without protective snow cover ticks can die of freezing under these conditions (Jore et al., 2014).

In each time step the sub model checks whether the minimum temperature *t_min_* is below a threshold value of minus 18.9 °C. If that is the case, a certain number of ticks of a cohort (determined by the freezing rate) are considered dead and are hence removed from the modelled population. Table 8 contains the parameters that belong to the freezing sub model.

**Table 8: Overview of temperature threshold and freezing rate in case of extreme cold.**

| **Parameter** | **Value** | **Unit** | **Reference** |
| --- | --- | --- | --- |
| FREEZING_RATE | 0.03 | 1/day | own estimation |
| FREEZING_MIN_TEMP_WITHOUT_SNOW | -18.9 | °C | Gray et al., 2009 |

**7.4 Activity**

The sub model *Activity* is responsible for controlling the activity status of the modelled tick population. It is implemented in the class Activity. Activity in context of this model means that ticks are either questing (also known as host-seeking) or inactive unless they are engorged. The change from one activity state to the other depends on the microclimate to which the tick is exposed. When temperatures and relative humidity are suitable, unfed ticks begin to quest (Perret et al., 2000). They do this by climbing up the vegetation to find a host. To prevent desiccation, ticks must return to the ground, where the relative humidity is greater than the relative humidity at host-seeking height due to the ground moisture (Randolph et al., 2004).

In each time step, the activity sub model checks the prevailing conditions in terms of temperature and relative humidity. When microclimatic conditions are suitable for questing, i.e. when temperature and humidity are within certain ranges (see Table 9), the number of questing ticks increases with a specific activation rate and the number of inactive ticks is reduced accordingly. Under suboptimal but possible conditions, only a very small proportion of ticks will quest. In this case the number of questing ticks increases at a much lower activation rate. In case the microclimatic conditions are unsuitable for host-seeking, the ticks will retreat to the ground to rehydrate with the default activation rate. The ticks are then classified as *inactive*. Tick activity is a dynamic process, i.e. the total number of ticks questing at a given time step is a result of the balance of activation (towards the questing state) and deactivation (towards the inactive stage) over the previous time steps. Table 9 contains an overview over the activation parameters values belonging to the activity sub model.

**Table 9: Overview of questing activation rates and temperature threshold values.**

| **Parameter** | **Value** | **Unit** | **Type** | **Reference** |
| --- | --- | --- | --- | --- |
| ACTIVATION_NECESSARY_MAXIMAL_MAX_TEMP | 35.0 | °C | float | MacLeod, 1935  Gray et al., 2016 |
| ACTIVATION_NECESSARY_MINIMAL_MAX_TEMP | 1.9 | °C | float | Perret et al., 2000 |
| ACTIVATION_NECESSARY_MINIMAL_MEAN_TEMP | 1.2 | °C | float | Perret et al., 2000 |
| ACTIVATION_NECESSARY_MINIMAL_HUMIDITY | 45.0 | °C | float | Greenfield, 2011 |
| ACTIVATION_OPTIMAL_MINIMAL_MAX_TEMP | 10.5 | °C | float | Perret et al., 2000 |
| ACTIVATION_OPTIMAL_MAXIMAL_MAX_TEMP | 26.0 | °C | float | Greenfield, 2011 |
| ACTIVATION_OPTIMAL_MINIMAL_MEAN_TEMP | 6.0 | °C | float | Gilbert et al., 2014 |
| ACTIVATION_OPTIMAL_MAXIMAL_MEAN_TEMP | 20.0 | °C | float | Kubiak & Dziekonska-Ryno, 2006 |
| OPTIMAL_SHARE_OF_ACTIVATION_RATE | 1.0 | 1 / day | float | - |
| SUBOPTIMAL_SHARE_OF_ACTIVATION_RATE | 0.05 | 1 / day | float | own estimation |
| activationRate | 0.02 | 1 / day | float | Determined by optimisation |

**7.5 Feeding**

The sub model *Feeding* controls the feeding success of ticks and includes a translocation process by which the ticks are moved to another location on the model landscape. It is implemented in the class *Feeding*. Feeding in the context of this model means that questing ticks find a host, attach themselves to it for their blood meal and drop off later at another location due to the movement of the host (Medlock et al., 2013). The feeding process takes place instantaneously. Neither the individual steps of this process nor the host animals are explicitly modelled.

In each time step, ticks feed across all cells of the model landscape at a specific rate that depends on their respective life cycle stage (see Table 10). We assume that the probability of a tick moving a certain distance is negatively correlated with the distance length (see Table 11). At the end of the feeding process ticks are considered engorged and the numbers in the respective cohorts are adjusted accordingly. Late feeders that find a host in or after late summer, i.e. approx. Mid-September do not develop to the next life cycle stage stage until the following year (O. Kahl 2020, pers. comm.). To prevent them from developing to the next life cycle stage after mid-September in an ongoing simulation, they are assigned to a separate cohort. Table 10 gives an overview over the feeding parameters of the feeding sub model. The distance probabilities of the translocation process can be found in Table 11.

**Table 10: Overview of feeding rates for each life cycle stage**

| **Parameter** | **Life Cycle Stage** | **Value** | **Unit** | **Type** | **Reference** |
| --- | --- | --- | --- | --- | --- |
| FEEDING_RATE | Larva | 0.01 | 1 / day | float | own estimation |
|  | Nymph | 0.03 | 1 / day | float | own estimation |
|  | Adult | 0.05 | 1 / day | float | own estimation |
| LATE_FEEDING_TIME | - | 232 | - | int |  |

**Table 11: Overview of the distance-dependent probabilities of the translocation process.** The distance is given in number of cells away from the current cell.

| **Distance** | 1 | 2 | 3 | 4 | 5 | 6 | 7 | 8 | 9 |
| --- | --- | --- | --- | --- | --- | --- | --- | --- | --- |
| **Probability** | 0.25 | 0.25 | 0.20 | 0.15 | 0.05 | 0.04 | 0.03 | 0.02 | 0.01 |

**7.6. Weather**

The sub model *weather* controls the processing of weather data, i.e. daily mean, minimum and maximum temperature and daily relative humidity. This sub model is implemented in the class Weather. During model initialisation (see Section 5), the weather data for temperature and relative humidity are read in from a CSV file from the model input folder and internally stored in array lists. During model execution the corresponding values of a day are then retrieved at each time step. These values must then be adapted to the microclimatic conditions depending on the season of the year and the specific habitat type. The reason for this adjustment is that measured values from a weather station or simulated values from a climate simulation do not represent the actual conditions on the ground to which ticks are exposed. For example, during summer the air temperatures in a forest is cooler than above canopy or in clearings (Geiger et al., 1995; Bonan, 2016). Also the actual relative humidity in the litter layer of a forest differs considerably from measured values from nearby weather stations (Boehnke et al., 2017). Therefore, these deviations must be corrected accordingly. The season and habitat dependent adjustment values used by our model can be found in Table 12.

**Table 12: Overview of adjustment values to adapt weather and climate data to the real conditions at the ground where ticks live.**

| **Parameter** | **Season** | **Habitat** | **Value** | **Unit** | **Reference** |
| --- | --- | --- | --- | --- | --- |
| Temperature | Spring / Autumn | Pasture | 0.0 | °C | own estimation |
|  |  | Ecotone | -1.0 | °C | own estimation |
|  |  | Wood | -2.0 | °C | own estimation |
| Temperature | Summer | Pasture | 0.0 | °C | own estimation |
|  |  | Ecotone | -2.0 | °C | Geiger et al., 1995 |
|  |  | Wood | -4.0 | °C | Bonan, 2016 |
| Relative Humidity | All seasons | Pasture | 12 | % | own estimation |
|  |  | Ecotone | 18 | % | own estimation |
|  |  | Wood | 24 | % | Boehnke et al., 2017 |

**References**

Boehnke, D., Brugger, K., Pfäffle, M., Sebastian, P., Norra, S., Petney, T., Oehme, R., Littwin, N., Lebl, K., Raith, J., Walter, M., Gebhardt, R., & Rubel, F. (2015). Estimating ixodes ricinus densities on the landscape scale. International Journal of Health Geographics, 14(1), 23. https://doi.org/10.1186/s12942-015-0015-7

Boehnke, D., Gebhardt, R., Petney, T., & Norra, S. (2017). On the complexity of measuring forests microclimate and interpreting its relevance in habitat ecology: The example of ixodes ricinus ticks. Parasites & Vectors, 10(1), 549. https://doi.org/10.1186/s13071-017-2498-5

Bonan, G. B. (2016). Ecological climatology: Concepts and applications (3. ed.). Cambridge University Press. https://doi.org/10.1017/CBO9781107339200

Brugger, K., Boehnke, D., Petney, T., Dobler, G., Pfeffer, M., Silaghi, C., Schaub, G. A., Pinior, B., Dautel, H., Kahl, O., Pfister, K., Süss, J., & Rubel, F. (2016). A density map of the tick-borne encephalitis and lyme borreliosis vector ixodes ricinus (acari: Ixodidae) for germany. Journal of Medical Entomology, 53(6), 1292–1302. https://doi.org/10.1093/jme/tjw116

Brugger, K., Walter, M., Chitimia-Dobler, L., Dobler, G., & Rubel, F. (2018). Forecasting next season’s ixodes ricinus nymphal density: The example of southern germany 2018. Experimental and Applied Acarology, 75(3), 281–288. https://doi.org/10.1007/s10493-018-0267-6

Cayol, C., Koskela, E., Mappes, T., Siukkola, A., & Kallio, E. R. (2017). Temporal dynamics of the tick ixodes ricinus in northern europe: Epidemiological implications. Parasites & Vectors, 10(1), 166. https://doi.org/10.1186/s13071-017-2112-x

Clement, J., Vercauteren, J., Verstraeten, W. W., Ducoffre, G., Barrios, J. M., Vandamme, A.-M., Maes, P., & van Ranst, M. (2009). Relating increasing hantavirus incidences to the changing climate: The mast connection. International Journal of Health Geographics, 8, 1. https://doi.org/10.1186/1476-072X-8-1

Costa, A., & Soares, A. (2009). Homogenization of climate data: Review and new perspectives using geostatistics. Mathematical Geosciences, 41, 291–305. https://doi.org/10.1007/s11004-008-9203-3

Geiger, R., Aron, R. H., & Todhunter, P. (1995). The climate near the ground (Fifth Edition). Vieweg+Teubner Verlag. https://doi.org/10.1007/978-3-322-86582-3

Gilbert, L., Aungier, J., & Tomkins, J. L. (2014). Climate of origin affects tick (ixodes ricinus) host-seeking behavior in response to temperature: Implications for resilience to climate change? Ecology and evolution, 4(7), 1186–1198. https://doi.org/10.1002/ece3.1014

Gray, J. S. (1991): The development and seasonal activity of the tick Ixodes ricinus: a vector of Lyme borreliosis. In: Review of Medical and Veterinary Entomology 79 (6), S. 323–333.

Gray, J. S., Dautel, H., Estrada-Peña, A., Kahl, O., Lindgren, E., & Fries, B. (2009). Effects of climate change on ticks and tick-borne diseases in europe. Interdisciplinary Perspectives on Infectious Diseases, 2009, 593232. https://doi.org/10.1155/2009/593232

Gray, J. S., Kahl, O., Lane, R. S., Levin, M. L., & Tsao, J. I. (2016). Diapause in ticks of the medically important ixodes ricinus species complex. Ticks and Tick-borne Diseases, 7 (5), 992–1003. https://doi.org/10.1016/j.ttbdis.2016.05.006

Greenfield, B. P. J. (2011). Environmental parameters affecting tick (ixodes ricinus) distribution during the summer season in richmond park, london. Bioscience Horizons, 4(2), 140–148. https://doi.org/10.1093/biohorizons/hzr016

Grimm, V., Berger, U., DeAngelis, D. L., Polhill, J. G., Giske, J., & Railsback, S. F. (2010). The odd protocol: A review and first update. Ecological Modelling, 221(23), 2760–2768. https://doi.org/10.1016/j.ecolmodel.2010.08.019

Grimm, V., Railsback, S. F., Vincenot, C. E., Berger, U., Gallagher, C., DeAngelis, D. L., Edmonds, B., Ge, J., Giske, J., & Groeneveld, J. (2020). The odd protocol for describing agent-based and other simulation models: A second update to improve clarity, replication, and structural realism. Journal of Artificial Societies and Social Simulation, 23(2).

Hauser, G., Rais, O., Morán Cadenas, F., Gonseth, Y., Bouzelboudjen, M., & Gern, L. (2018). Influence of climatic factors on ixodes ricinus nymph abundance and phenology over a long-term monthly observation in switzerland (2000-2014). Parasites & Vectors, 11(1), 289. https://doi.org/10.1186/s13071-018-2876-7

Jacob, D., Petersen, J., Eggert, B., Alias, A., Christensen, O. B., Bouwer, L. M., Braun, A., Colette, A., Déqué, M., Georgievski, G., Georgopoulou, E., Gobiet, A., Menut, L., Nikulin, G., Haensler, A., Hempelmann, N., Jones, C., Keuler, K., Kovats, S., … Yiou, P. (2014). Euro-cordex: New high-resolution climate change projections for european impact research. Regional Environmental Change, 14(2), 563–578. https://doi.org/10.1007/s10113-013-0499-2

Jore, S., Vanwambeke, S. O., Viljugrein, H., Isaksen, K., Kristoffersen, A. B., Woldehiwet, Z., Johansen, B., Brun, E., Brun-Hansen, H., Westermann, S., Larsen, I.-L., Ytrehus, B., & Hofshagen, M. (2014). Climate and environmental change drives ixodes ricinus geographical expansion at the northern range margin. Parasites & Vectors, 7, 11. https://doi.org/10.1186/1756-3305-7-11

Konnert, M., Schneck, D., & Zollner, A. (2016). Blühen und Fruktifizieren unserer Waldbäume in den letzten 60 jahren. LWF Wissen, 74, 37–45.

Kubiak, K., & Dziekonska-Rynko, J. (2006). Seasonal activity of the common european tick, ixodes ricinus [linnaeus, 1758], in the forested areas of the city of olsztyn and its sorroundings. Wiadomości Parazytologiczne, 52(1).

Lindström, A., & Jaenson, T. G. (2003). Distribution of the common tick, ixodes ricinus (acari: Ixodidae), in different vegetation types in southern sweden. Journal of Medical Entomology, 40(4), 375–378. https://doi.org/10.1603/0022-2585-40.4.375

MacLeod, J. (1935). Ixodes ricinus in relation to its physical environment. Parasitology, 27 (1), 123–144. https://doi.org/10.1017/S0031182000015006

Maraun, D. (2016). Bias correcting climate change simulations - a critical review. Current Climate Change Reports, 2(4), 211–220. https://doi.org/10.1007/s40641-016-0050-x

Medlock, J. M., Hansford, K. M., Bormane, A., Derdakova, M., Estrada-Peña, A., George, J.-C., Golovljova, I., Jaenson, T. G. T., Jensen, J.-K., Jensen, P. M., Kazimirova, M., Oteo, J. A., Papa, A., Pfister, K., Plantard, O., Randolph, S. E., Rizzoli, A., Santos-Silva, M. M., Sprong, H., … van Bortel, W. (2013). Driving forces for changes in geographical distribution of ixodes ricinus ticks in europe. Parasites & Vectors, 6(1), 1. https://doi.org/10.1186/1756-3305-6-1

Ostfeld, R. S., & Brunner, J. L. (2015). Climate change and ixodes tick-borne diseases of humans. Philosophical Transactions of the Royal Society B: Biological Sciences, 370(1665), 20140051. https://doi.org/10.1098/rstb.2014.0051

Perret, J.-L., Guigoz, E., Rais, O., & Gern, L. (2000). Influence of saturation deficit and temperature on ixodes ricinus tick questing activity in a lyme borreliosis-endemic area (switzerland). Parasitology Research, 86(7), 554–557. https://doi.org/10.1007/s004360000209

Randolph, S. E. (2004). Tick ecology: Processes and patterns behind the epidemiological risk posed by ixodid ticks as vectors. Parasitology, 129 Suppl, S37–65. https://doi.org/10.1017/s0031182004004925

Ribeiro, S., Caineta, J., & Costa, A. C. (2016). Review and discussion of homogenisation methods for climate data. Physics and Chemistry of the Earth, Parts A/B/C, 94, 167–179. https://doi.org/10.1016/j.pce.2015.08.007

Schulz, M., Mahling, M., & Pfister, K. (2014). Abundance and seasonal activity of questing ixodes ricinus ticks in their natural habitats in southern germany in 2011. Journal of Vector Ecology, 39(1), 56–65. https://doi.org/10.1111/j.1948-7134.2014.12070.x

Teutschbein, C., & Seibert, J. (2012). Bias correction of regional climate model simulations for hydrological climate-change impact studies: Review and evaluation of different methods. Journal of Hydrology, 456-457, 12–29. https://doi.org/10.1016/j.jhydrol.2012.05.052
