# Supplementary material for: Model-based extrapolation of ecological systems under future climate scenarios: The example of Ixodes ricinus ticks
Source: PLoS One. 2022 Apr 22;17(4):e0267196. doi: 10.1371/journal.pone.0267196 (PMC9032420; doi:10.1371/journal.pone.0267196)
Supplement: S2 Appendix — (DOCX) [file pone.0267196.s002.docx]

**Appendix S2 - Results of the IRIS model calibration**


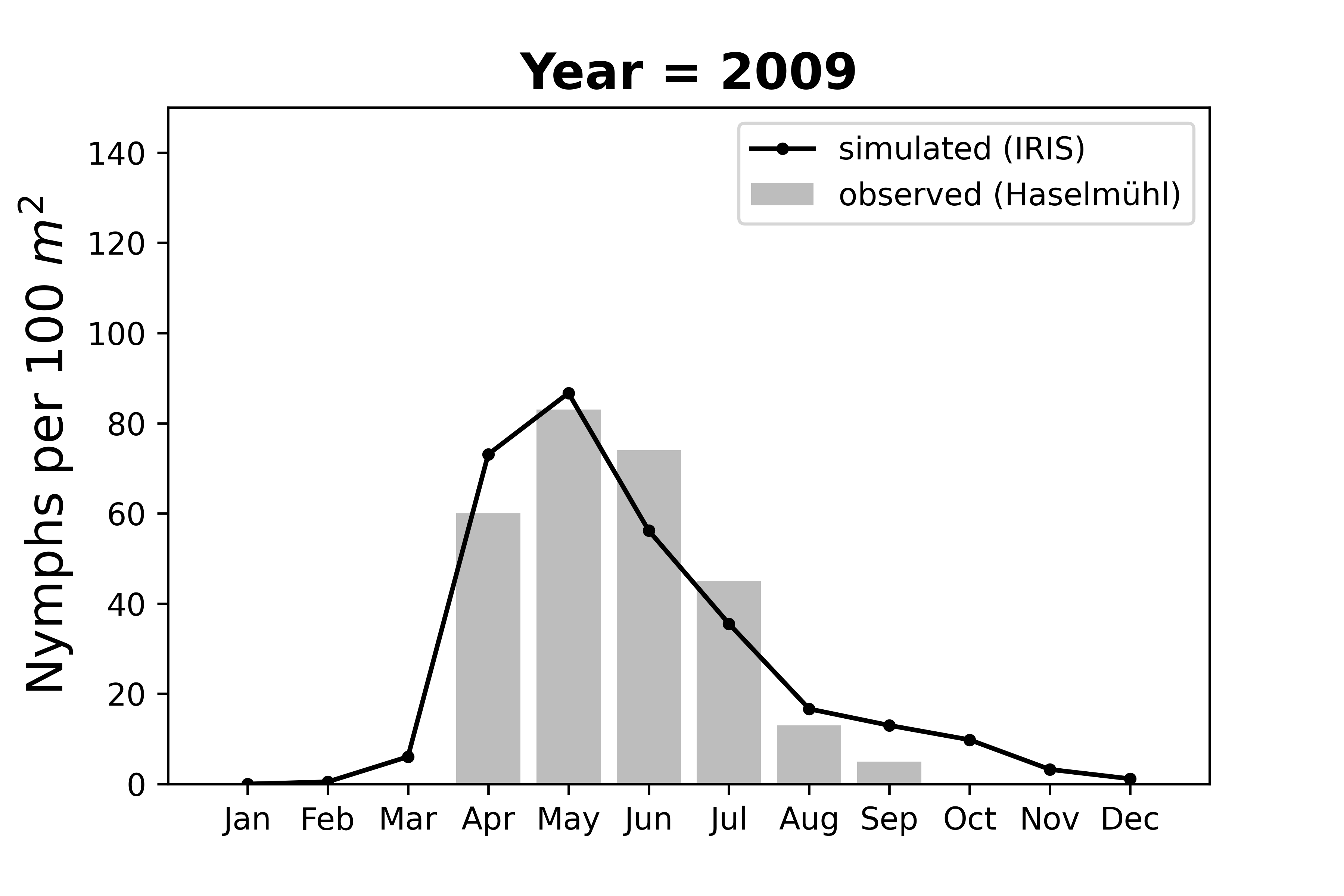


**Fig 1. Result of the model calibration for the year 2009.** The grey bars show the monthly observed nymphal densities (nymphs / 100 m^2^) at the sampling site in Haselmühl. The black connected dots show the monthly simulated nymphal densities with calibrated parameter values. The root-mean-square error (RMSE) is 9.29.


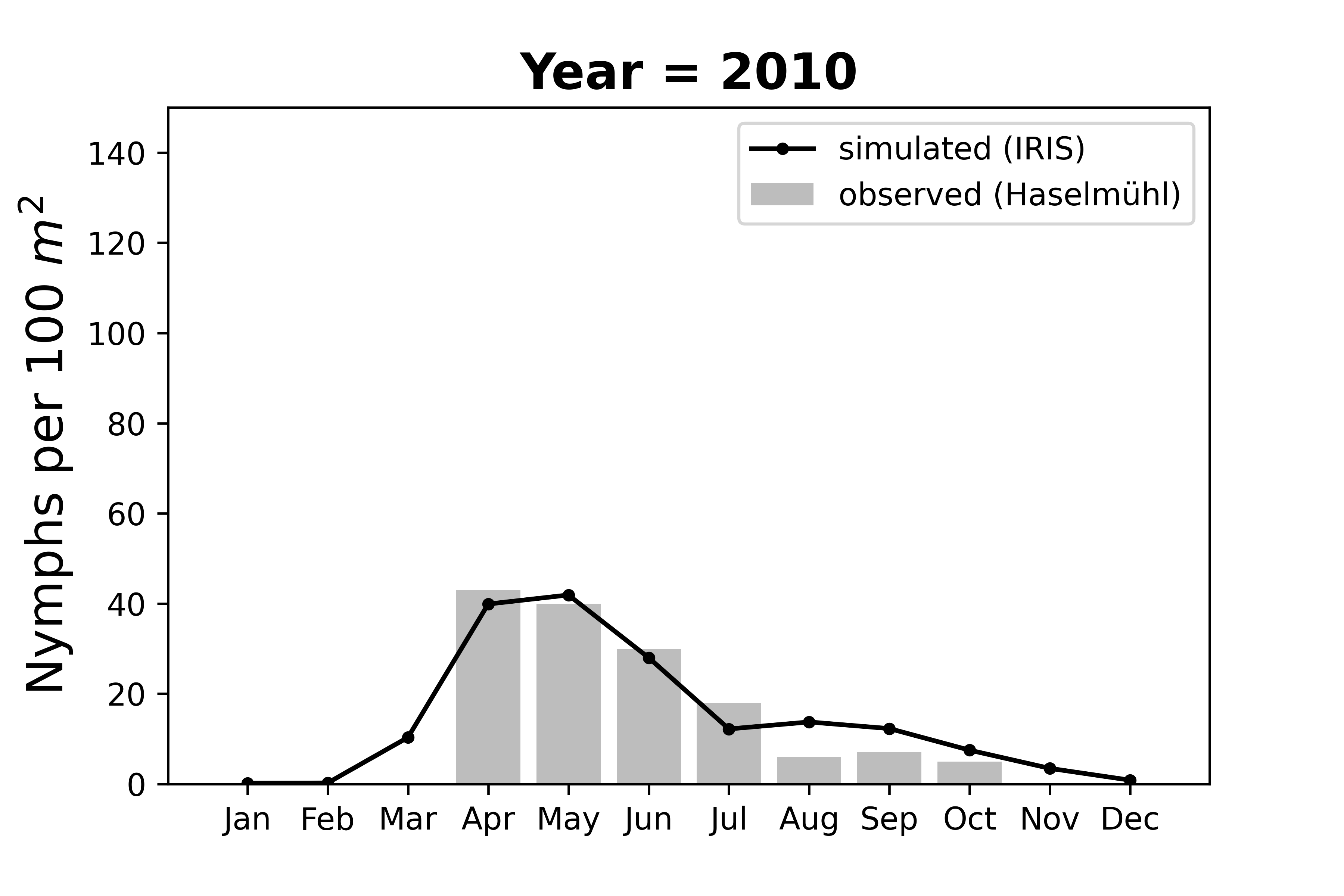


**Fig 2. Result of the model calibration for the year 2010.** The grey bars show the monthly observed nymphal densities (nymphs / 100 m^2^) at the sampling site in Haselmühl. The black connected dots show the monthly simulated nymphal densities with calibrated parameter values. The root-mean-square error (RMSE) is 4.70.


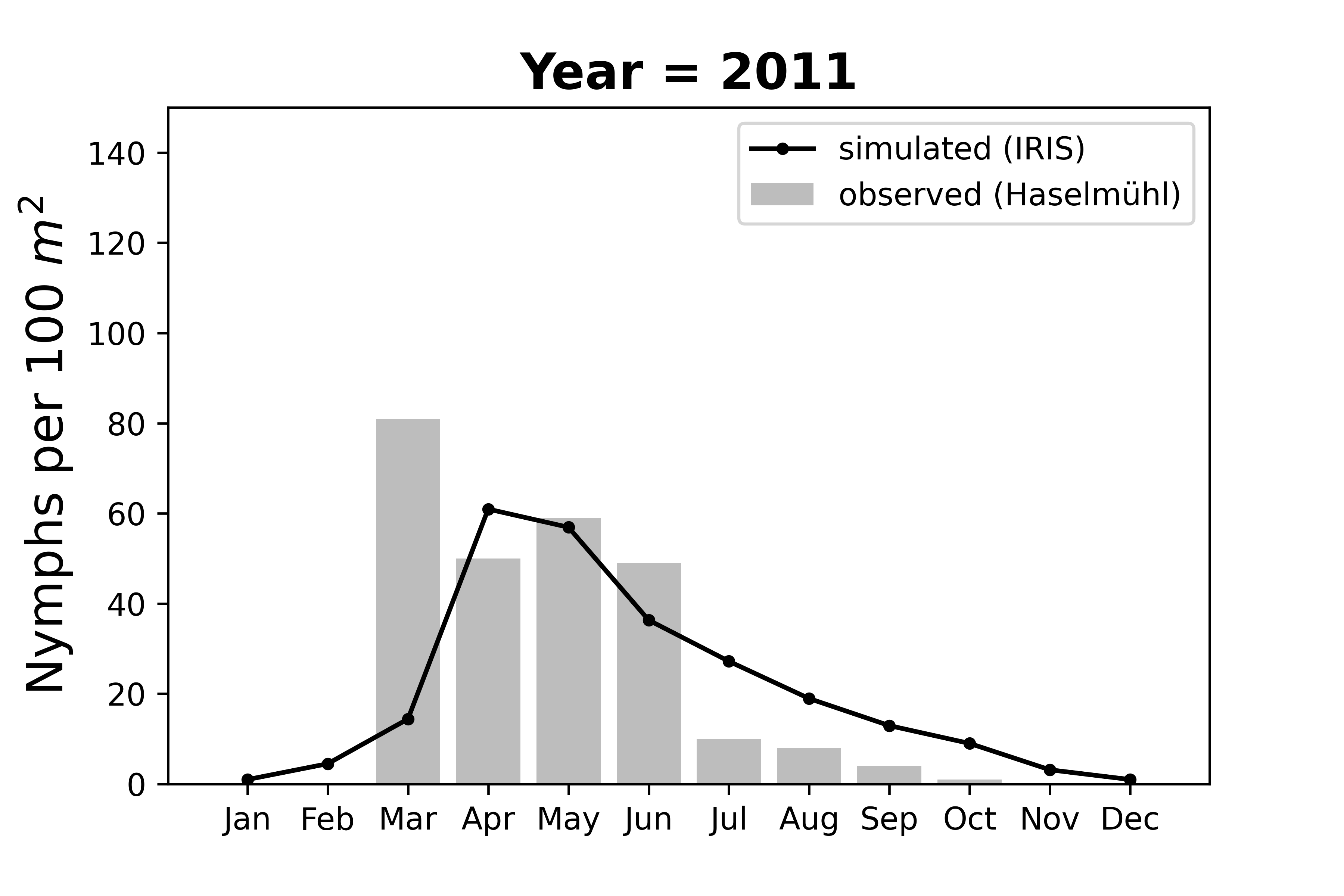


**Fig 3. Result of the model calibration for the year 2011.** The grey bars show the monthly observed nymphal densities (nymphs / 100 m^2^) at the sampling site in Haselmühl. The black connected dots show the monthly simulated nymphal densities with calibrated parameter values. The root-mean-square error (RMSE) is 21.03.


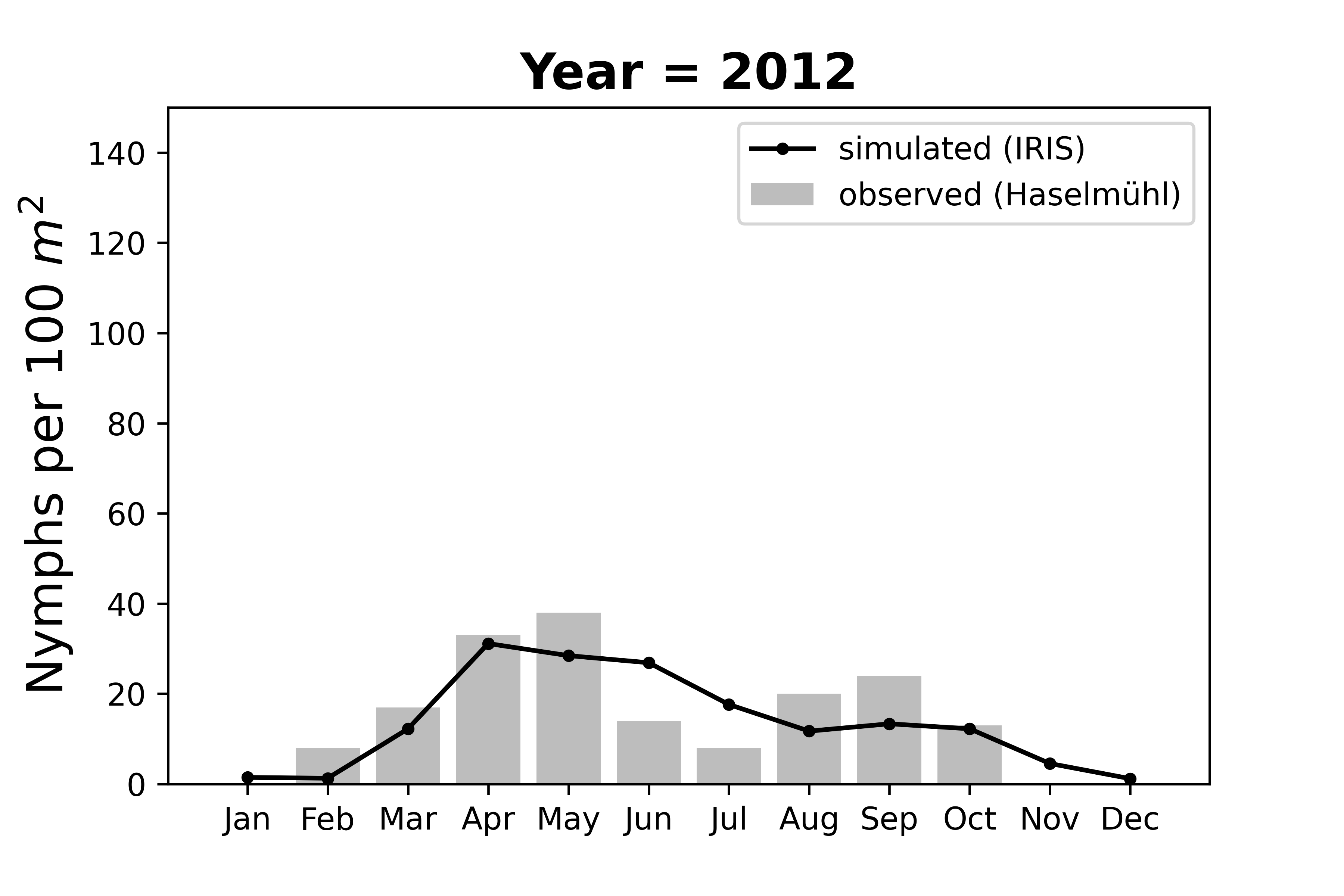


**Fig 4. Result of the model calibration for the year 2012.** The grey bars show the monthly observed nymphal densities (nymphs / 100 m^2^) at the sampling site in Haselmühl. The black connected dots show the monthly simulated nymphal densities with calibrated parameter values. The root-mean-square error (RMSE) is 7.23.


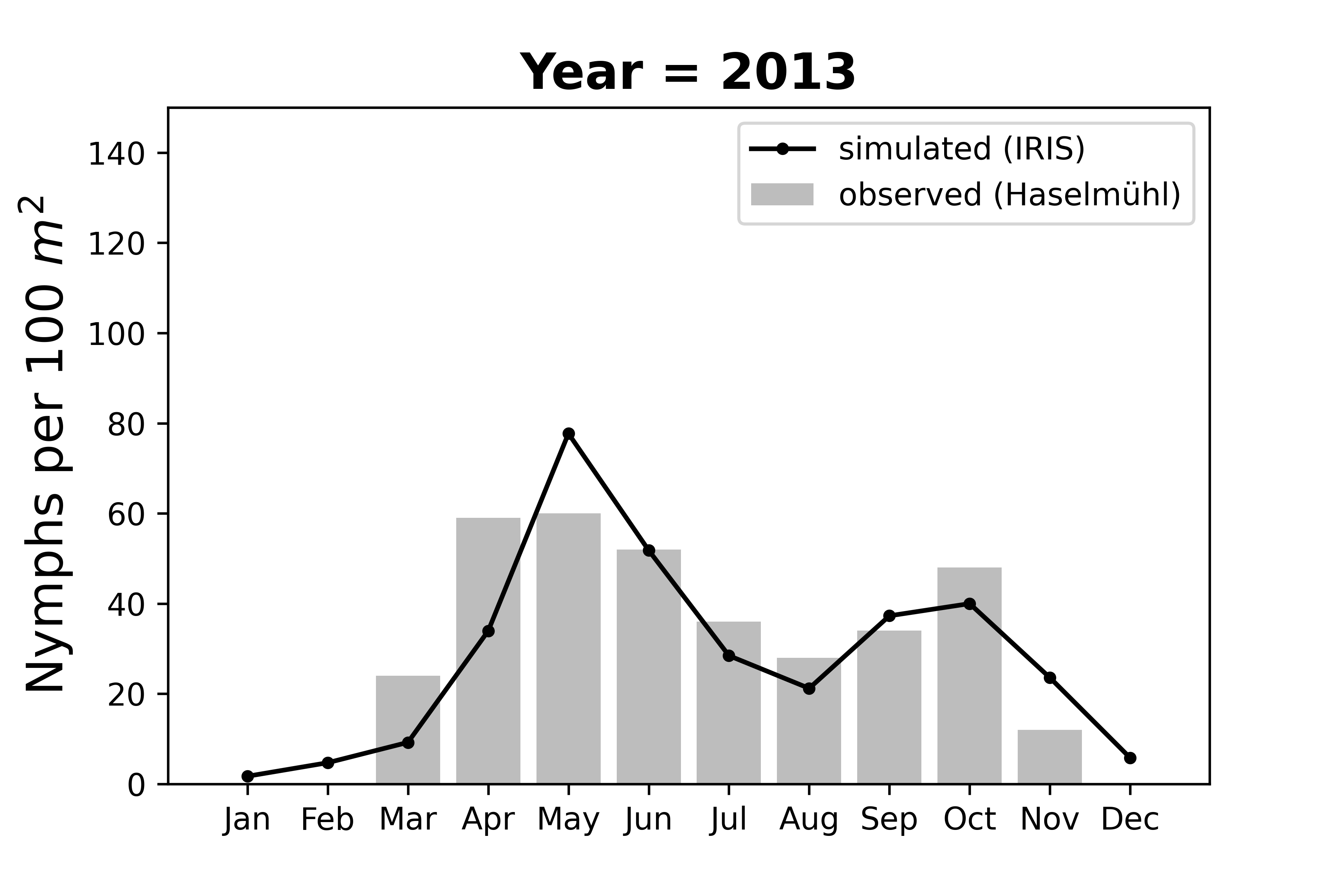


**Fig 5. Result of the model calibration for the year 2013.** The grey bars show the monthly observed nymphal densities (nymphs / 100 m^2^) at the sampling site in Haselmühl. The black connected dots show the monthly simulated nymphal densities with calibrated parameter values. The root-mean-square error (RMSE) is 11.29.


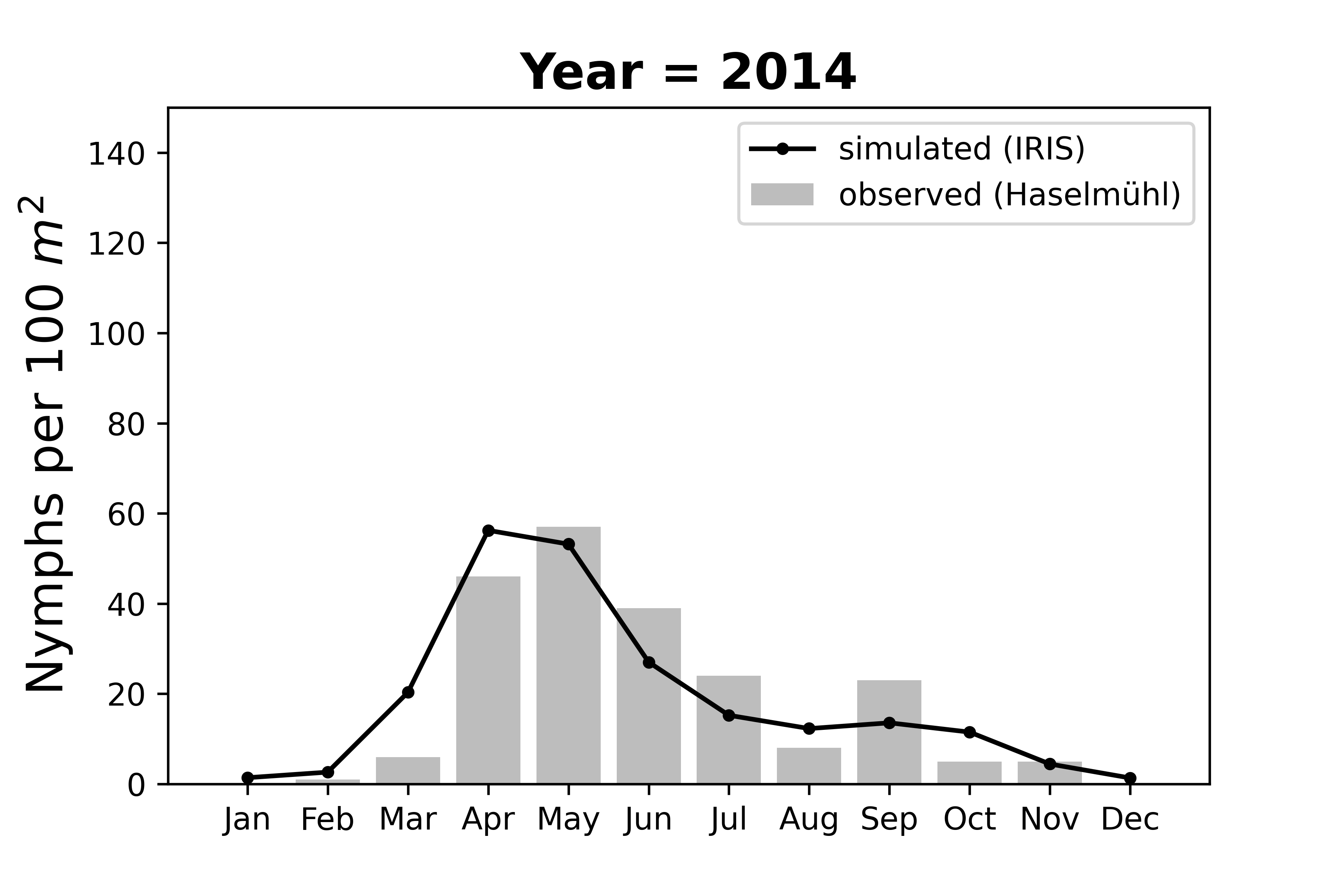


**Fig 6. Result of the model calibration for the year 2014.** The grey bars show the monthly observed nymphal densities (nymphs / 100 m^2^) at the sampling site in Haselmühl. The black connected dots show the monthly simulated nymphal densities with calibrated parameter values. The root-mean-square error (RMSE) is 7.65.


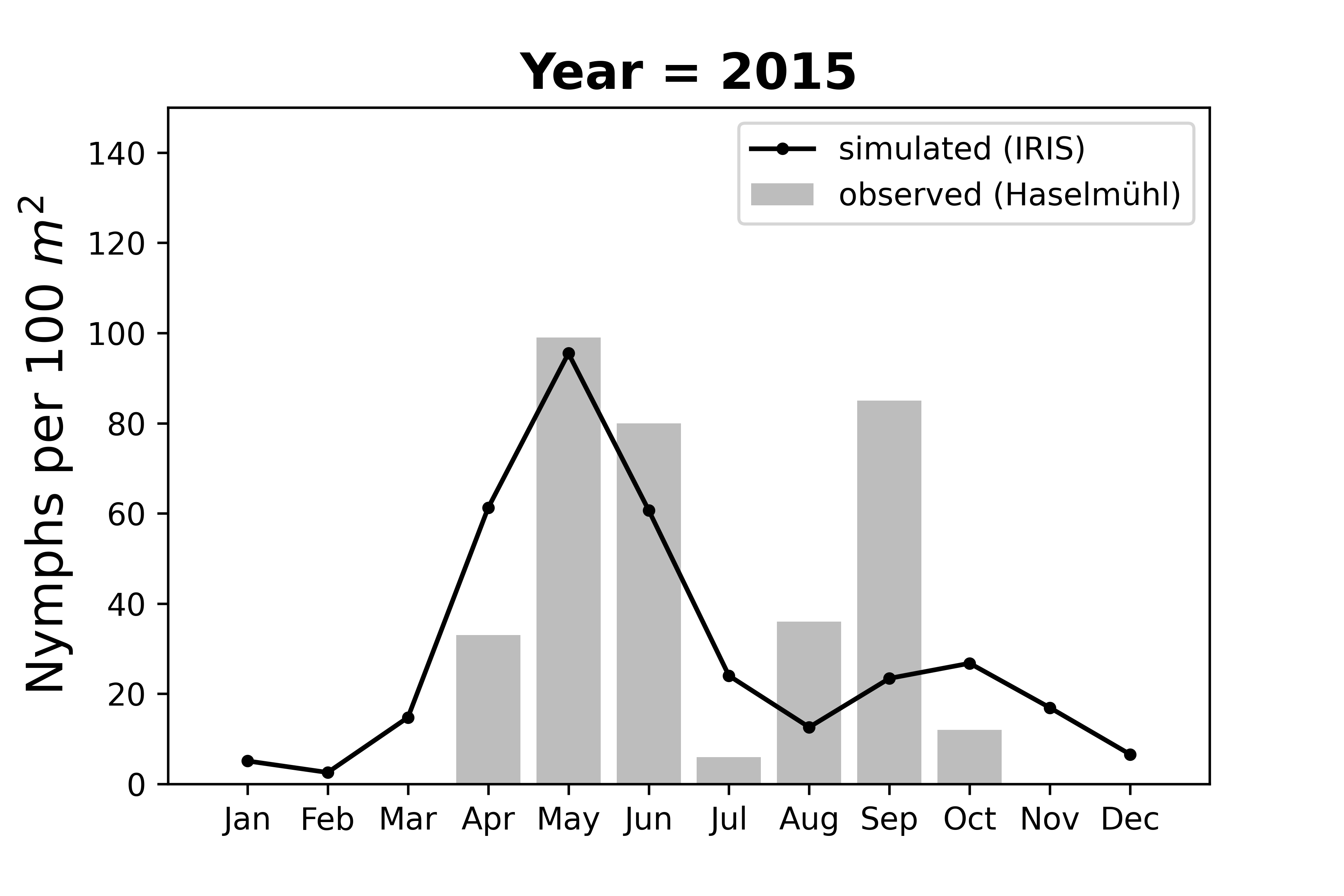


**Fig 7. Result of the model calibration for the year 2015.** The grey bars show the monthly observed nymphal densities (nymphs / 100 m^2^) at the sampling site in Haselmühl. The black connected dots show the monthly simulated nymphal densities with calibrated parameter values. The root-mean-square error (RMSE) is 23.53.


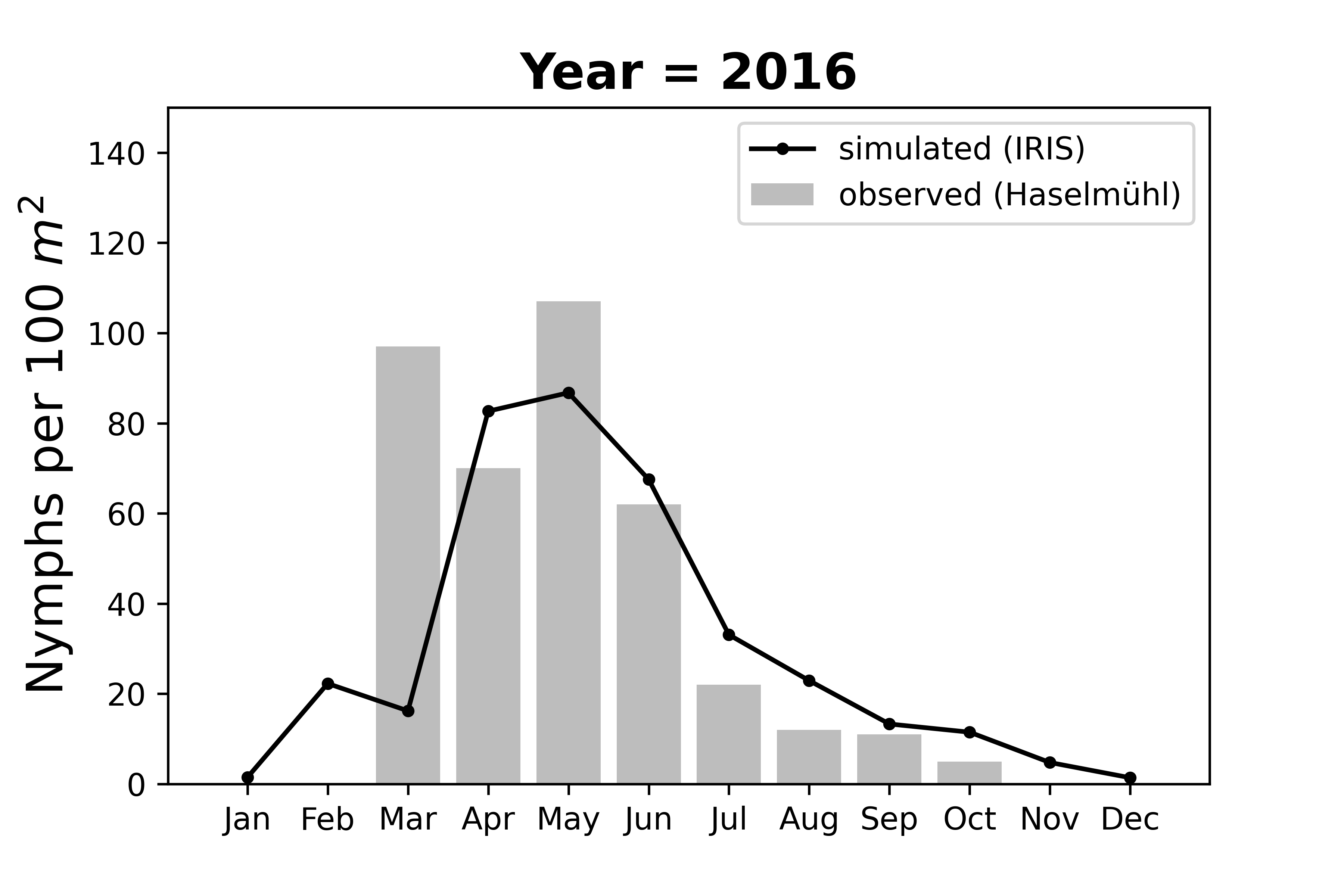


**Fig 8. Result of the model calibration for the year 2016.** The grey bars show the monthly observed nymphal densities (nymphs / 100 m^2^) at the sampling site in Haselmühl. The black connected dots show the monthly simulated nymphal densities with calibrated parameter values. The root-mean-square error (RMSE) is 25.72.


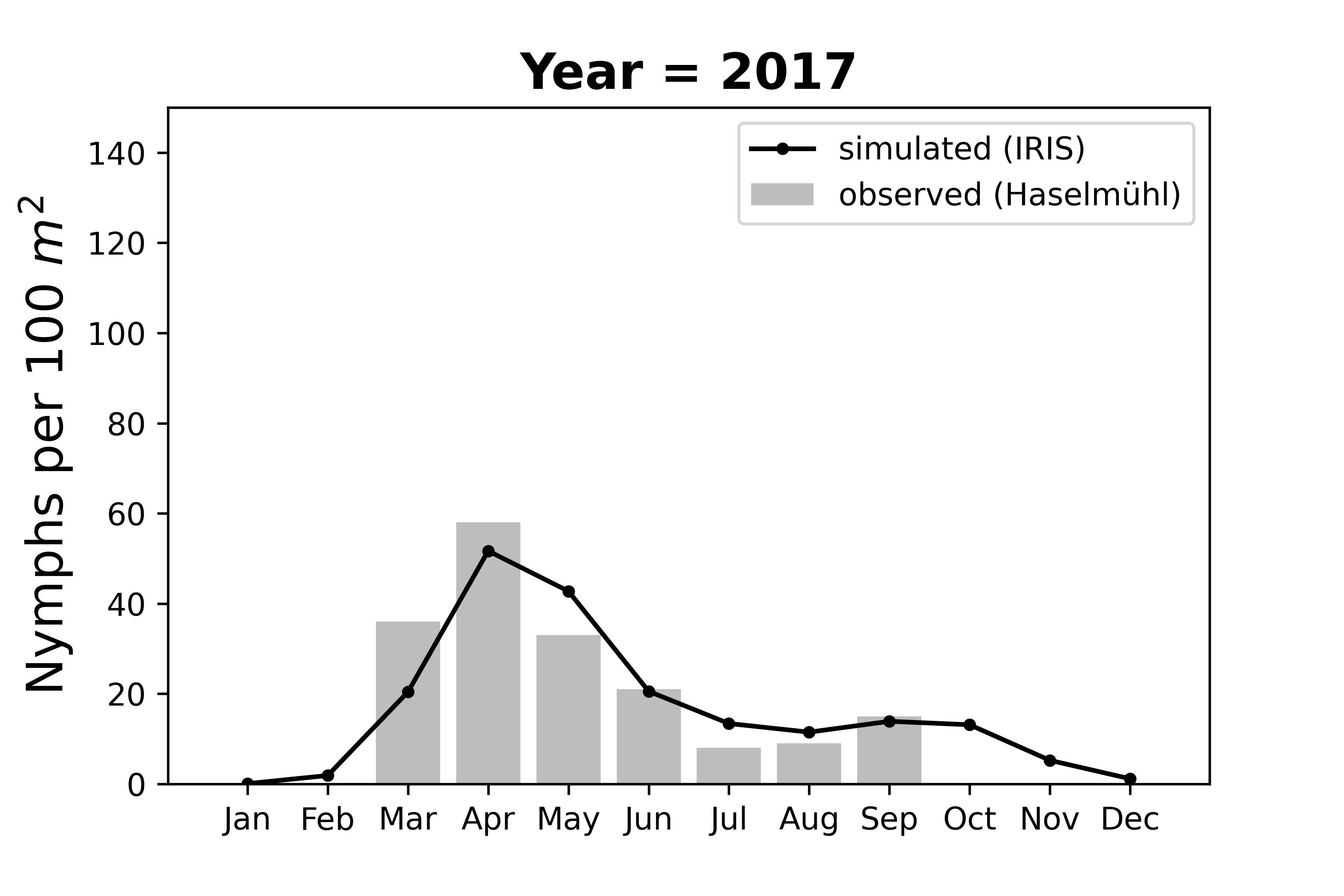


**Fig 9. Result of the model calibration for the year 2017.** The grey bars show the monthly observed nymphal densities (nymphs / 100 m^2^) at the sampling site in Haselmühl. The black connected dots show the monthly simulated nymphal densities with calibrated parameter values. The root-mean-square error (RMSE) is 6.82.


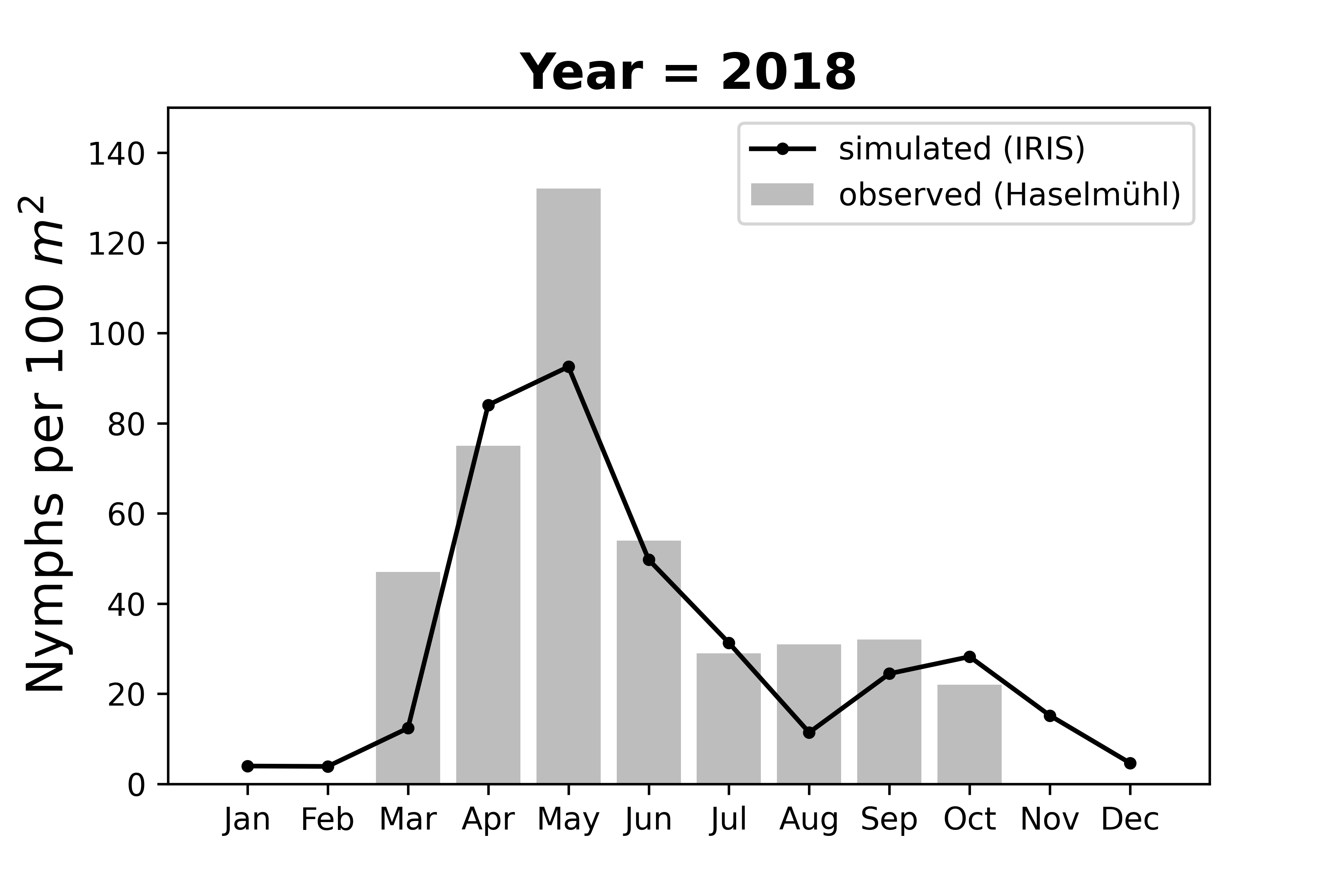


**Fig 10. Result of the model calibration for the year 2018.** The grey bars show the monthly observed nymphal densities (nymphs / 100 m^2^) at the sampling site in Haselmühl. The black connected dots show the monthly simulated nymphal densities with calibrated parameter values. The root-mean-square error (RMSE) is 20.44.
