# Supplementary material for: Model-based extrapolation of ecological systems under future climate scenarios: The example of Ixodes ricinus ticks
Source: PLoS One. 2022 Apr 22;17(4):e0267196. doi: 10.1371/journal.pone.0267196 (PMC9032420; doi:10.1371/journal.pone.0267196)
Supplement: S3 Appendix — (DOCX) [file pone.0267196.s003.docx]

**Appendix S3 - Derived quantities contrasted with the annual mean temperature, the annual median temperature and years on the x-axis.**


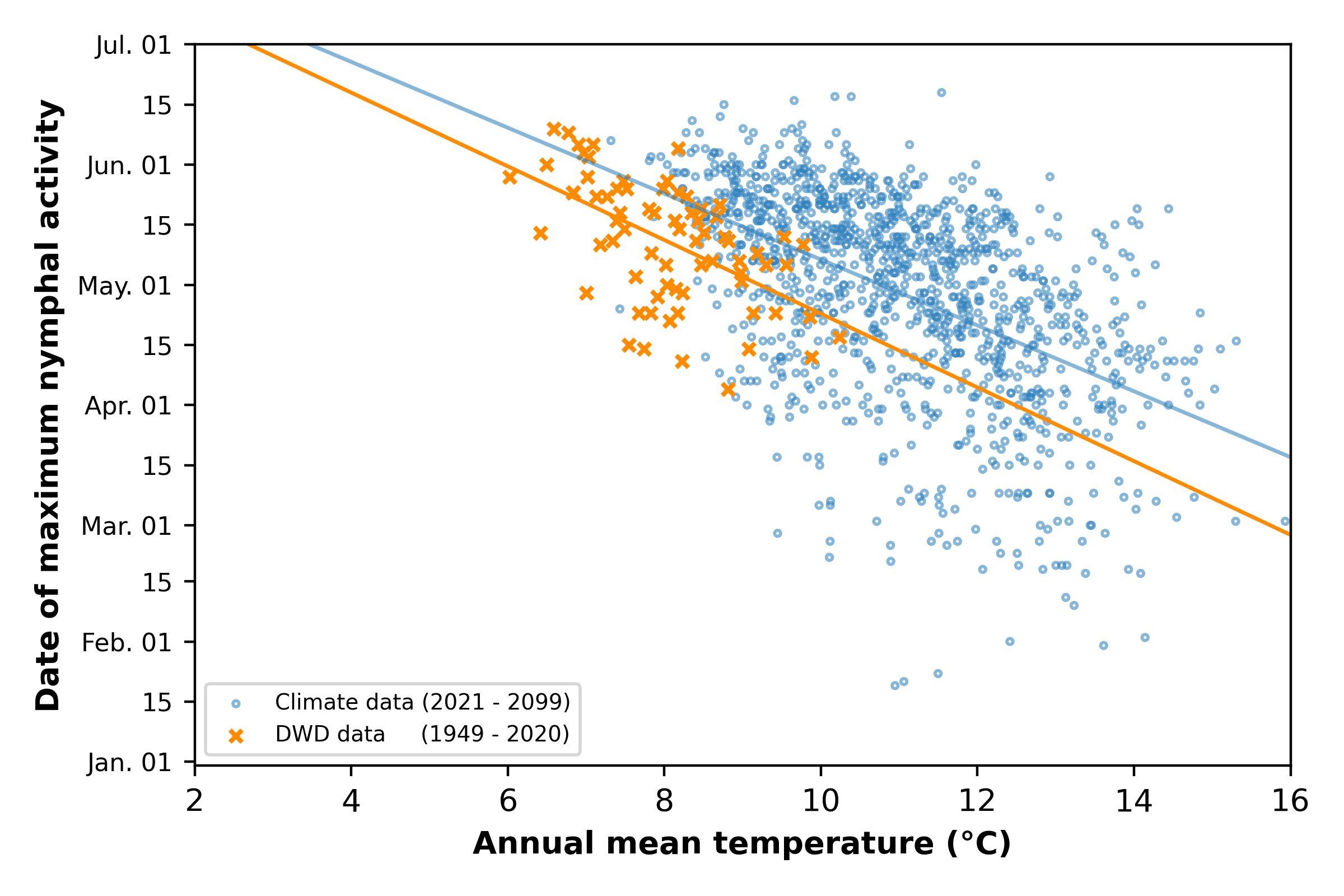


**Fig 1. Relationship of the annual mean temperature between January and June (x-axis) and the date of the maximum number of questing nymphs on the model landscape (y-axis).**


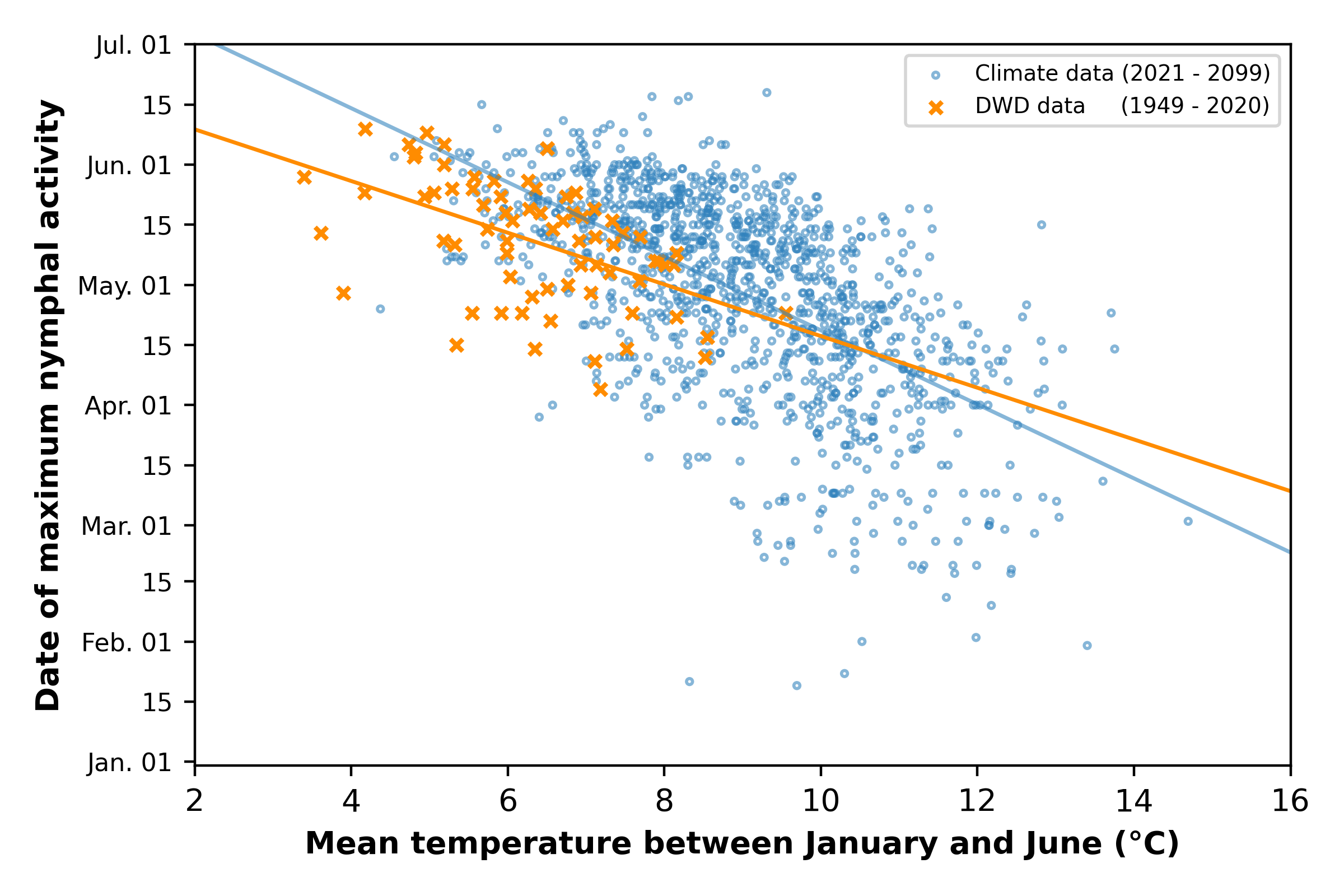


**Fig 2. Relationship of the mean temperature between January and June (x-axis) and the date of the maximum number of questing nymphs on the model landscape (y-axis).**


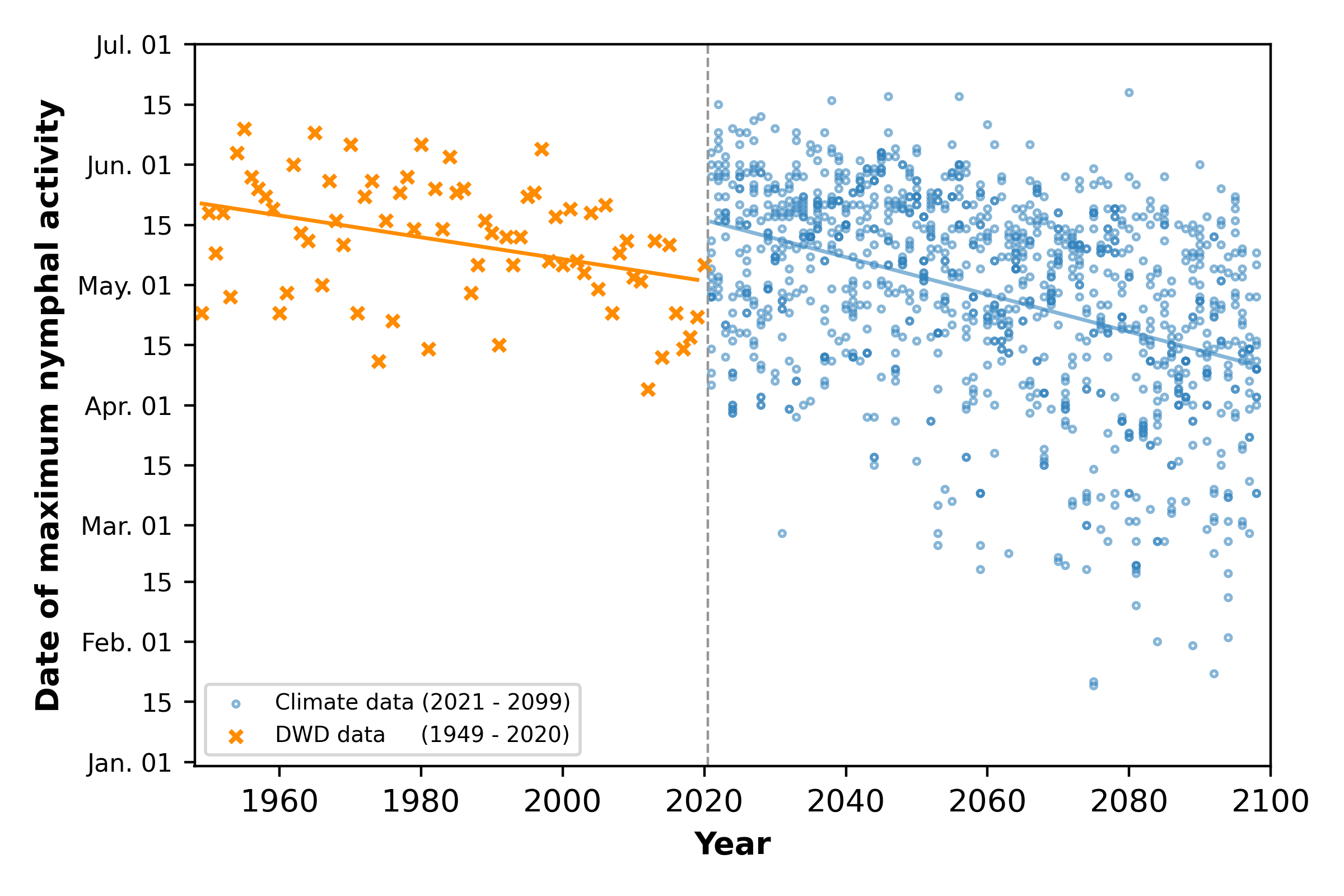


**Fig 3. Relationship of the year (x-axis) and the date of the maximum number of questing nymphs on the model landscape (y-axis).**


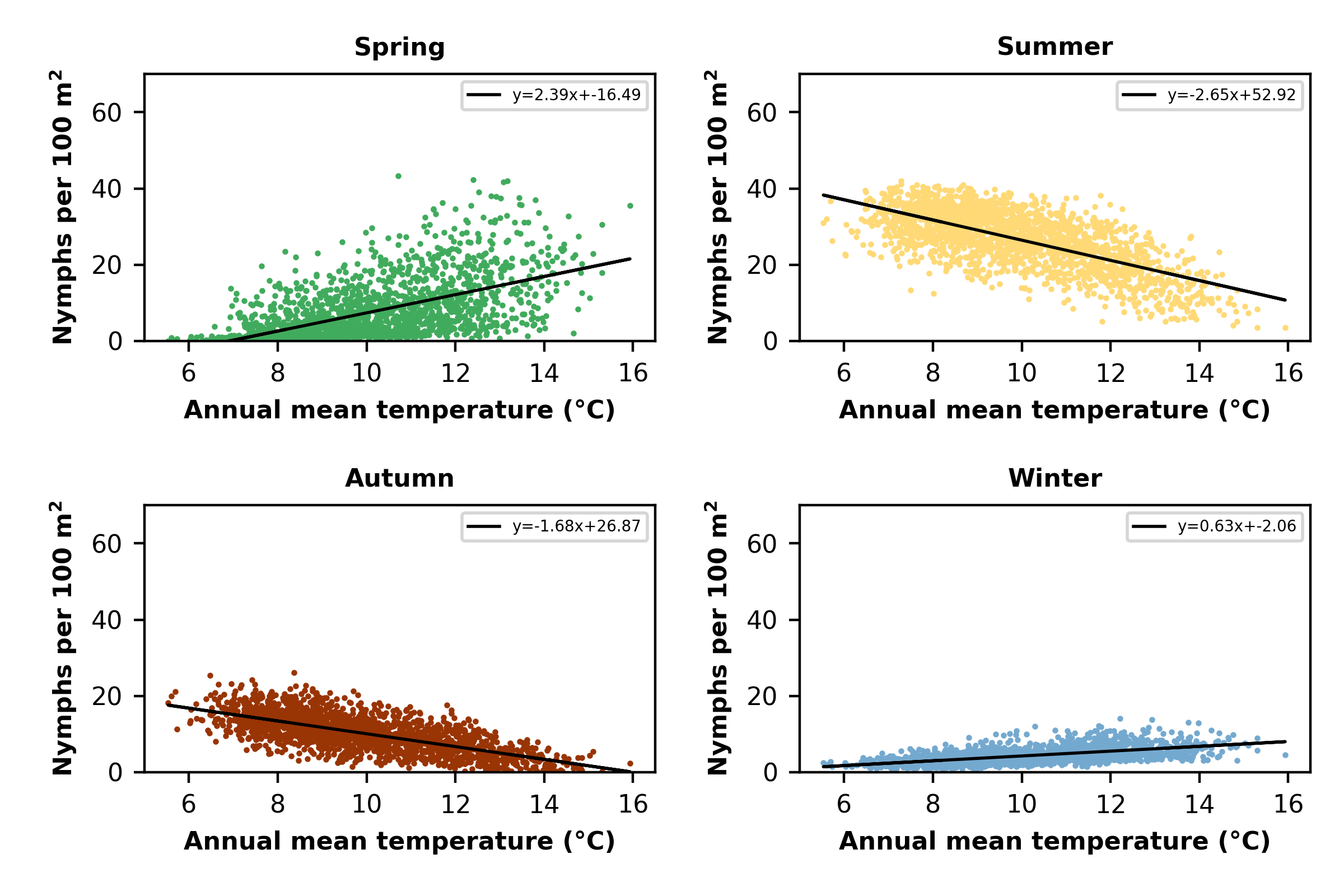


**Fig 4. Relationship of the annual mean temperature (x-axis) and the density of questing nymphs / 100 m^2^ (y-axis) for the seasons of a year.**


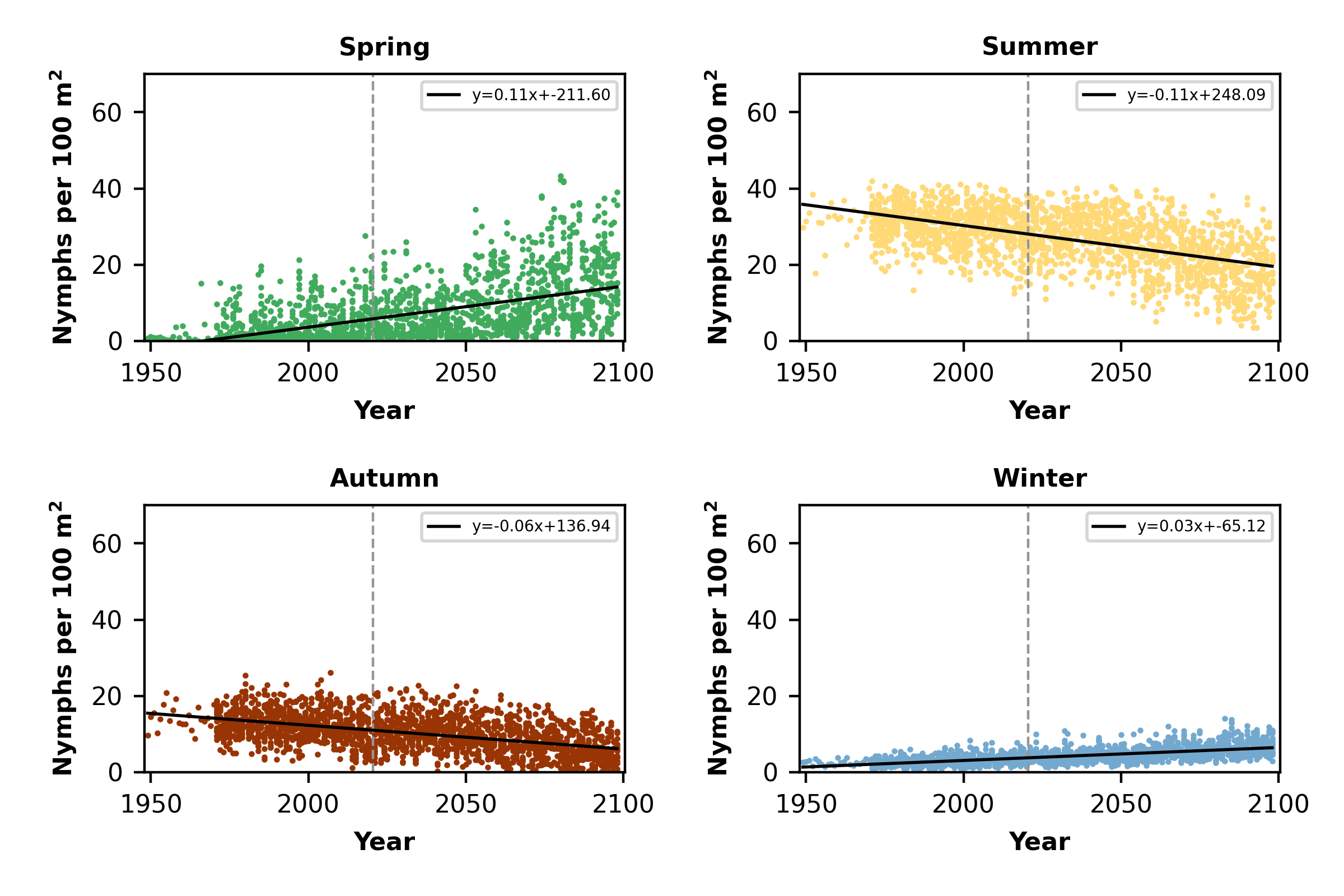


**Fig 5. Relationship of the year (x-axis) and the density of questing nymphs / 100 m^2^ (y-axis) for the seasons of a year.**
